# Operando Raman spectroscopy uncovers hydroxide and CO species enhance ethanol selectivity during pulsed CO_2_ electroreduction

**DOI:** 10.1038/s41467-024-48052-3

**Published:** 2024-05-11

**Authors:** Antonia Herzog, Mauricio Lopez Luna, Hyo Sang Jeon, Clara Rettenmaier, Philipp Grosse, Arno Bergmann, Beatriz Roldan Cuenya

**Affiliations:** 1 Department of Interface Science, Fritz Haber Institute of the Max-Planck Society, 14195 Berlin, Germany; 2 Present Address: Massachusetts Institute of Technology, Research Laboratory of Electronics, 77 Massachusetts Ave, Cambridge, MA 02139 USA; 3 Present Address: Chemical Sciences Division, Lawrence Berkeley National Laboratory, Berkeley, CA 94720 USA; 4 Present Address: Korea Institute of Science and Technology, 5 Hwarang-ro 14-gil, Wolgok 2(i)-dong, Seongbuk-gu, Seoul, South Korea

**Keywords:** Electrocatalysis, Electrocatalysis

## Abstract

Pulsed CO_2_ electroreduction (CO_2_RR) has recently emerged as a facile way to in situ tune the product selectivity, in particular toward ethanol, without re-designing the catalytic system. However, in-depth mechanistic understanding requires comprehensive operando time-resolved studies to identify the kinetics and dynamics of the electrocatalytic interface. Here, we track the adsorbates and the catalyst state of pre-reduced Cu_2_O nanocubes ( ~ 30 nm) during pulsed CO_2_RR using sub-second time-resolved operando Raman spectroscopy. By screening a variety of product-steering pulse length conditions, we unravel the critical role of co-adsorbed OH and CO on the Cu surface next to the oxidative formation of Cu-O_ad_ or CuO_x_/(OH)_y_ species, impacting the kinetics of CO adsorption and boosting the ethanol selectivity. However, a too low OH_ad_ coverage following the formation of bulk-like Cu_2_O induces a significant increase in the C_1_ selectivity, while a too high OH_ad_ coverage poisons the surface for C-C coupling. Thus, we unveil the importance of co-adsorbed OH on the alcohol formation under CO_2_RR conditions and thereby, pave the way for improved catalyst design and operating conditions.

## Abstract (web summary)

Controlling the selectivity of the electroreduction of CO_2_ to value-added products is challenging. Here, authors use sub-second operando Raman spectroscopy to analyze Cu catalyst surfaces and discover the pivotal role of co-adsorbed OH and CO in enhancing ethanol selectivity.

## Introduction

Within the scope of reducing global CO_2_ emissions to limit climate change, carbon net-zero technologies have gained enormous interest. One promising technology is the CO_2_RR, which closes the carbon cycle by using renewable energies to transform CO_2_ back into useful chemicals and fuels^1^. Among the metals studied, only copper electrodes have the unique ability to produce the desired energy-dense alcohols and hydrocarbons such as ethanol and ethylene in significant amounts^2^. However, for further commercialization in high-current electrolyzers, Cu-based catalysts still suffer from a broad selectivity distribution, low activity, and low stability during long-term operation.

Several strategies have been developed to address these issues and to increase the product distribution toward C_2+_ products, which include tuning the catalyst structure and composition^3–7^ and modifying the electrolyte^8–10^. Additionally, the (initial) oxidation state of Cu plays a major role, particularly oxidized Cu species and oxide-derived Cu materials showed a major improvement in the selectivity and stability of the catalysts^11–16^. Thus, a simple way to regenerate the desired oxidation state of Cu in situ is by means of pulsed potential CO_2_RR, where an electrocatalytic potential alternates between an oxidizing and a CO_2_RR potential^17^. Hereby, the key pulse parameters are the applied cathodic and anodic potential, the pulse shape as well as the pulse length^17^.

By varying only the cathodic and anodic pulse lengths, while keeping the other pulse parameters constant, the catalytic properties of Cu electrodes could be enhanced^17–19^. In this way, the amount, as well as the type of Cu oxide species (Cu^+^, Cu^2+^) formed on the catalyst surface, was controlled^20,21^. In particular, distorted non-crystalline Cu(I)/Cu(II) domains were formed at short anodic pulses (below 2 s) as evidenced by operando X-ray absorption spectroscopy (XAS) and X-ray diffraction (XRD), which had been associated with an enhanced ethanol formation^18^. The dynamic alternation of the potential impacts also the restructuring (faceting and defects) and the roughening of the catalyst at longer anodic pulse lengths (above 1 s), which were observed to enhance the selectivity of either methane or ethylene^18,20,22^. However, the induced dynamics on the surface coverage of hydrogen (H_ad_), hydroxide (OH_ad_), and carbon monoxide (CO_ad_) are crucial to fully understand the underlying principle of selectivity changes via pulsed CO_2_RR. Several studies suggested that the application of the anodic pulse leads to higher OH coverage, while H_ad_ is removed due to the positive polarization of the electrodes^19,21,23–26^. The resulting higher OH coverage was proposed to stabilize the active CO_atop_ compared to the inactive CO_bridge_ species^27–29^, which then enhances the ethanol selectivity. The beneficial effect of a higher OH coverage and the resulting higher local pH was also linked to a change in the CO adsorption energy as well as a lower C–C coupling barrier^30^. While the CO binding configuration was previously tracked with operando surface-enhanced infrared absorption spectroscopy (SEIRAS)^27,29^, there is to date no experimental evidence for the postulated changes in the OH and CO coverages, and the selectivity-determining role of the adsorbed OH beyond CO dimerization stays unclear. Moreover, it remains challenging to determine the possible presence of surface Cu oxides due to the lack of surface-sensitive operando characterizations^1^. Thus, these tools are needed to gain a better mechanistic comprehension of pulsed CO_2_RR.

In this study, we, therefore, applied operando sub-second time-resolved surface-enhanced Raman spectroscopy (SERS), which is an ideal method to simultaneously track the changes in the surface oxidation state of Cu and the changes in the OH_ad_ and CO_ad_ surface adsorbates as well as their surface coverage. In this way, we followed the impact of the applied anodic or cathodic pulse lengths during pulsed CO_2_RR. Thus, square-wave potential pulses were alternated between an electrocatalytic cathodic potential *E*_c_ at −1.0 V and an oxidizing anodic potential *E*_a_ at +0.6 V versus the reversible hydrogen electrode (RHE). Pre-reduced Cu_2_O nanocubes (NCs) served as catalysts, which exhibit tunable selectivities for valuable C_2+_ products such as ethanol or C_1_ products such as methane, depending on the selected pulse length^18^. In particular, we correlated the enhancement of ethanol observed to an increase of the OH_ad_ versus CO_ad_ coverage and the formation of Cu-O_ad_ and CuO_*x*_/(OH)_*y*_ species under selected pulsed CO_2_RR conditions. Here, we demonstrate the impact of the adsorbates and surface species on the obtained selectivity trends in dependence on the applied pulse lengths to gain novel mechanistic insights in a sub-second time-resolved way.

## Results

### Time-dependent evolution of SERS

**Fig. 1:**
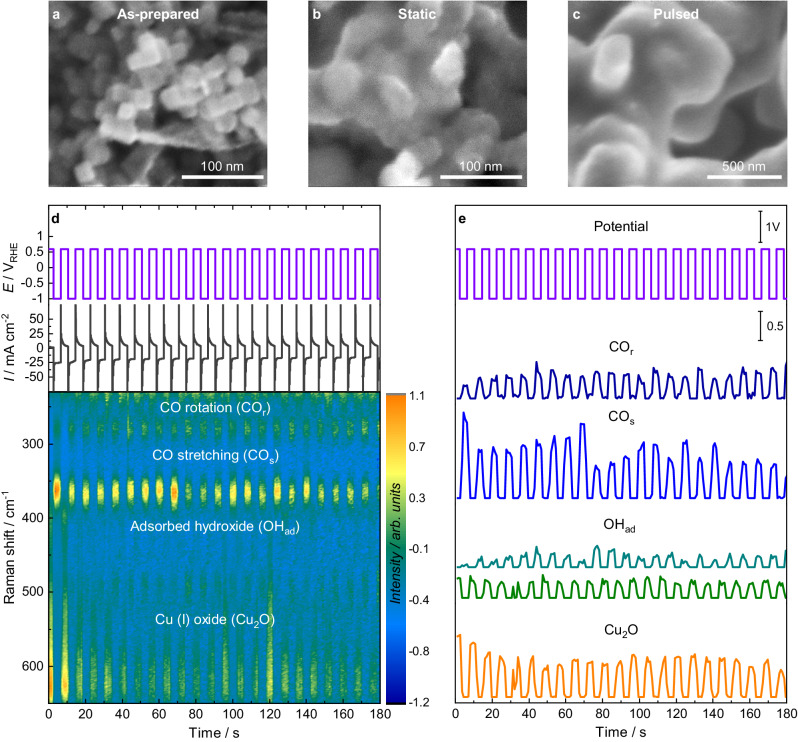
Morphology and temporal evolution of the SERS intensity during pulsed CO_2_RR. **a** Ex situ SEM images of the as-prepared electrodes, **b** after potentiostatic CO_2_RR at -1.0 V for 1 h, and **c** after pulsed CO_2_RR at *E*_c_ = −1.0 V and *E*_a_ = 0.6 V with *t*_c_ = *t*_*a*_ = 4 s for 1 h. **d** Applied potential and current density over time with *t*_c_ = *t*_a_ = 4 s (top) and corresponding temporal evolution of the SERS signal intensity with marked Raman bands (bottom). **e** Applied potential over time and intensities of fits of selected characteristic Raman bands, namely the Cu–CO rotation (CO_r_, 280 cm^−1^, dark blue), Cu–CO stretching (CO_s_, 360 cm^−1^, light blue), Cu–OH_ad_ (OH_ad_, 495 cm^−1^ at −1.0 V in turquoise and 450 cm^−1^ at +0.6 V in green) and the copper(I) oxide (Cu_2_O, sum of 530 and 620 cm^−1^ divided by two, orange) bands.

Figure 1a shows a scanning electron microscopy (SEM) image of the Cu_2_O NC (~20 nm in size) pre-catalysts deposited on a glassy carbon electrode. The pre-catalyst was first pre-reduced in an electrochemical operando Raman flow cell setup (Supplementary Note 1, Supplementary Figs. 1, 2), resulting in loss of the initial cubic shape and the growth of the particles (~30 nm in size, Fig. 1b). Subsequently, pulsed CO_2_RR with a selected cathodic pulse length *t*_c_ and anodic pulse length *t*_a_ was applied (Supplementary Table 1). For all pulsed CO_2_RR measurements in this study, *E*_c_ = −1.0 V and *E*_a_ = +0.6 V (vs. RHE, for all shown potentials in this study), and the electrolyte consists of 0.1 M CO_2_-saturated potassium bicarbonate (KHCO_3_) if not stated differently. We note that the morphology of the particles changed to larger structures after pulsed CO_2_RR, as observed in the SEM image in Fig. 1c. Figure 1d presents the applied potential and the measured current (top) during the symmetric pulses with *t*_c_ = *t*_a_ = 4 s and the corresponding normalized SERS signal intensities over the Raman shift (bottom) as a function of the time. For the normalization of the SERS spectra, the intensity values were modified to a mean of 0 and a variance of 1. We note that the optical properties of the working catalyst are changing under potential pulse conditions as the reduced Cu_2_O nanocubes transform into rough, porous nanostructures. Therefore, to account for different SERS enhancement at different pulse length conditions, the SERS bands in this study were only compared to each other within the same spectra to obtain intensity ratios (Supplementary Note 2). The periodic responses of the characteristic SERS bands in this spectroscopic region were fitted in Fig. 1e (with fit examples in Supplementary Fig. 3). The selected characteristic bands here are:

(I) the CO_ad_ vibrations on Cu, namely the Cu–CO rotation (CO_r_) and stretching (CO_s_) vibrations at 280 and 360 cm^−1^, where CO is considered to be adsorbed on Cu as shown with surface characterization methods^31,32^. The CO bands appear during each cathodic pulse as CO_2_RR intermediates and disappear during the anodic pulses^6,33,34^. The relative CO surface coverage (CO_cov_) can be obtained by the intensity ratio of the two CO bands as CO_cov_ = Intensity (CO_s_)/Intensity (CO_r_). This relationship was derived in detail in our previous study through operando measurements in the presence of different CO concentrations and through DFT vibrational analysis for different CO coverages on Cu(100) surfaces^33^. However, the structural and morphological differences between the Cu(100) derived relationship to the oxide-derived Cu used in this work do not allow a direct comparison of the ratios and the CO coverage:
(II) the OH_ad_ vibration on or close to Cu, which can appear during the cathodic pulse at 495 cm^−1^ and during the anodic pulse redshifted at 450 cm^−1^ if OH is adsorbed or close to the Cu surface via dipole-dipole interactions^34–36^:
(III) the bands related to Cu_2_O on the surface at 410 cm^−1^ (multiphonon process), 530 cm^-1^ (Raman-allowed *F*_2g_ mode), and 620 cm^−1^ (IR-allowed *F*_1u_ mode), which appear during each anodic pulse due to the oxidation of Cu and disappear again during each cathodic pulse due to its reduction^6,34,35,37^. The additional presence of CuO or Cu(OH)_2_ as a minor component cannot be excluded due to similar band positions^38^.

While the assignment of CO_ad_ and Cu_2_O is straightforward, the assignment of OH_ad_ is still under debate, and some groups also assigned the band at 495 cm^−1^ to Cu–C and Cu–O species^35,39^. However, experiments in several previous studies with D_2_O showed a shift of the band position, which supports this assignment to chemisorbed OH_ad_ on Cu^35,36,40,41^. Furthermore, bands at ~500 and 700 cm^−1^, detectable during the electrochemical oxidation of Cu(111) and polycrystalline Cu surfaces, were linked by other groups to surface OH species through D_2_O experiments and DFT calculations^42^. Their DFT calculations propose that the 500 cm^−1^ band corresponds to a top-site OH stretching mode, possibly aligning with our scenario. In contrast, the 700 cm^−1^ band was attributed to the bending mode of free Cu–OH, a band mode that may be absent in our case due to co-adsorption with CO.

Additionally, a weak carbonate band at 1070 cm^−1^ was observed in some of the SERS spectra during the first 20 s, as shown in Supplementary Fig. 4^33,35,43^.

### Pulse length-dependent evolution of SERS

**Fig. 2:**
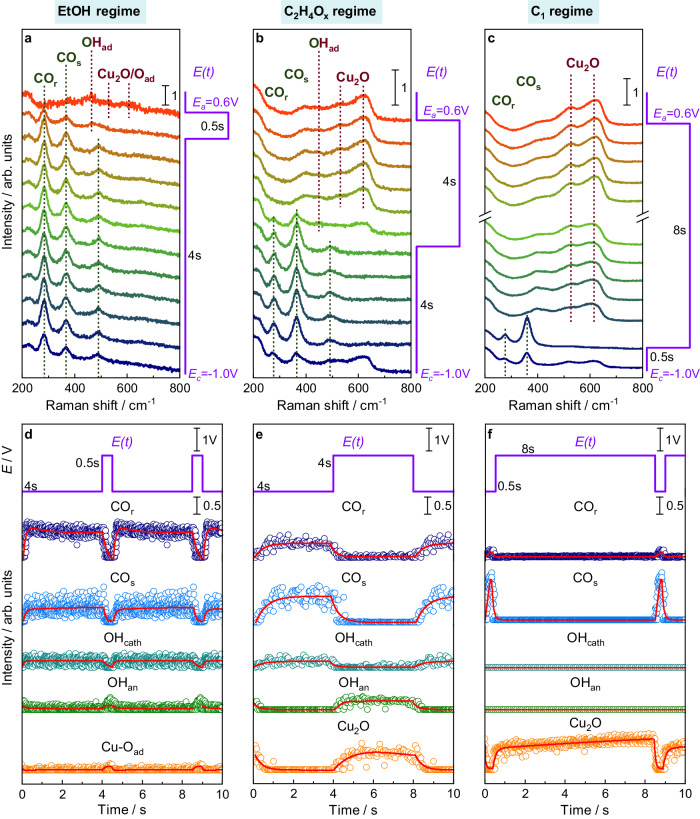
Pulse length-dependent evolution of SERS spectra and characteristic bands. **a**–**c** Normalized SERS spectra from bottom to top (as indicated with arrows) with highlighted characteristic SERS bands during pulsed CO_2_RR with varying pulse lengths at *E*_c_ = −1.0 V and *E*_a_ = +0.6 V and **d**–**f** intensities of fits of characteristic SERS bands averaged over one pulse sequence at selected pulse lengths. All averaged SERS spectra of **c** are shown in Supplementary Fig. 8. The data points in **d**–**f** represent the intensity fits of CO_r_ (280 cm^−1^, dark blue), CO_s_ (360 cm^-1^, light blue), OH_ad_ (495 cm^−1^ at −1.0 V in turquoise and 450 cm^-1^ at +0.6 V in green), Cu_2_O (sum of 530 and 620 cm^−1^ divided by two, orange) and Cu–O_ad_ (610 cm^−1^, orange) bands. The red lines denote the exponential fits and serve as guides for the eye.

In order to follow the impact of the applied cathodic and anodic pulse duration on the catalyst structure and the adsorbates, we averaged the normalized operando SERS spectra of selected pulse conditions and fitted the characteristic bands over one pulse sequence, as shown in Fig. 2. The emphasis is placed on three pulse regimes that are typical for the highest ethanol (EtOH regime, Fig. 2a, d), ethylene/acetaldehyde (C_2_H_4_O_*x*_ regime, Fig. 2b, e), and C_1_ product selectivity (C_1_ regime, Fig. 2c, f). The EtOH regime is characterized by short anodic pulses (*t*_a_ = 0.5 s), the C_2_H_4_O_*x*_ regime by intermediate anodic pulses (*t*_a_ = 4 s), and in both cases, the cathodic pulses have an intermediate length of 4 s. In contrast, long anodic pulses (*t*_a_ = 8 s) combined with short cathodic pulses (*t*_*c*_ = 0.5 s) represent the C_1_ regime^18^. Thereby, the intensity of the CO_s_ compared to the CO_r_ band decreased with shorter *t*_a_ and is the smallest in the EtOH regime. The OH_ad_ band is more intense during shorter *t*_a_ (0.5 s, 4 s) in the EtOH/C_2_H_4_O_*x*_ regime, while no OH_ad_ bands, but stronger Cu oxide bands during long *t*_a_ (8 s) and subsequent short *t*_c_ (0.5 s) could be detected in the C_1_ regime. These observations are underlined by additional SERS data at different pulse lengths (Supplementary Figs. 5, 6). During the anodic pulses, the Cu_2_O band intensities increased with the duration of the anodic pulse, being comparably weak at *t*_a_ = 0.5 s in the EtOH regime. Interestingly, there is also a contribution of Cu–O_ad_ visible in the region of 606–626 cm^−1^ during the anodic pulse in the EtOH regime for anodic pulses shorter than 2 s (Supplementary Fig. 5a, b)^42^. Under these conditions, also intense OH_ad_ bands and additional bands at 370–380 and 520–540 cm^−1^ were detected, which can be attributed to disordered CuO_*x*_/(OH)_*y*_ species^35,40^. Moreover, the carbonate band at 1070 cm^-1^ was predominantly observed at the onset of both the anodic and cathodic pulses, possibly linked to dynamic alterations of the electrical double layer. However, due to the low band intensities under these conditions, it is challenging to thoroughly analyze its characteristics further (Supplementary Figs. 5–7). To verify the reproducibility of the data the experiments of the main product regimes were repeated on freshly prepared electrodes showing similar spectra and trends (Supplementary Fig. 9).

To obtain additional insights into the average surface coverage of the characteristic species and their corresponding adsorption/desorption and oxidation/reduction kinetics, we performed exponential fits of the Raman band intensities during the averaged pulse profiles (Fig. 2d–f, Supplementary Fig. 10, Supplementary Tables 2–4). In particular, the kinetics of the ad- and desorption of CO on Cu altered with the applied pulse lengths (Supplementary Table 3). For example, in the EtOH regime, the CO_s_ band intensity increased twice as fast during the cathodic pulse as it decreased during the anodic pulse. Additionally, the CO_s_/CO_r_ band intensity ratio, which reflects the CO surface coverage, gradually and persistently escalated throughout the cathodic pulse (Supplementary Fig. 11). These findings suggest a change in the CO binding configuration during the cathodic pulse, which is expected to have implications for the catalytic function.

The differences in the CO binding configuration in terms of CO adsorption sites can be further evaluated by the C-O stretching vibration at higher wavenumbers (1900–2150 cm^−1^, Supplementary Figs. 12, 13). Particularly, the SERS spectra show the contribution of bridge CO (CO_bridge_ at 2030 cm^−1^) and two atop CO (CO_atop_) bands, namely the linear low-frequency CO (CO_LFB_ at 2065 cm^−1^) and high-frequency CO (CO_HFB_ at 2095 cm^−1^) bands, as described in the literature^29,44^. The contribution of CO_atop_ sites, which are usually related to C–C coupling^44^, is more prominent in the EtOH and C_2_H_4_O_x_ regimes (Supplementary Fig. 13). Instead, the contribution of CO_bridge_ sites, which are usually considered inactive for C–C coupling^27,29,44^, increased significantly in the C_1_ regime. Nevertheless, we found high uncertainties during several pulse sequences owing to the constantly evolving surface reactions that lead to the dynamic nature of the C–O stretching vibrations at high Raman shifts^34,44^.

For the OH_ad_ species, in turn, it is not easy to extract the ad- and desorption behavior since the bands additionally shifted upon the potential switch and partially overlapped with the evolving Cu_2_O bands during the anodic pulse (Fig. 2a, b). The shift of the bands probably resulted from the Stark effect due to the change in the electric field, with a reasonable Stark tuning rate of ~28 cm^−1^/V^45^, rather than from a change in the bonding configuration of OH_ad,_ as also discussed in the literature where the potential was only changed by 0.2 V^36^. Furthermore, it is likely that OH_ad_ is an intermediate of the Cu_2_O formation during the anodic pulse.

The adsorption of oxygen on the Cu surface and the oxidation to Cu_2_O over the anodic pulse was, under all applied pulse conditions, slower than the corresponding desorption/reduction upon the application of the cathodic pulse (Supplementary Table 4). In the C_1_ regime, the amount of surface Cu_2_O continuously increased the fastest, which indicates a continuous growth of the oxide layer over the course of the anodic pulse. Moreover, the spectroscopic weights of the three deconvoluted Cu_2_O bands changed at different applied pulse lengths (Supplementary Table 5). The relative intensity contribution of the band at 410 cm^−1^ is stronger during longer cathodic pulses, which may have been influenced by the higher ratio of OH_ad_^36^. On the other hand, the intensity of the 530 and 620 cm^−1^ bands contributes stronger for shorter cathodic pulses. Analyzing the time-dependent evolution of these bands in the C_1_ regime (Supplementary Fig. 14) revealed that the relative intensity of the band at 410 cm^−1^ was the highest in the first second of the anodic pulse, and its spectroscopic weight increased only slightly in the following. Also, the evolution of the intensity of the 620 cm^-1^ band suggests a temporary maximum in the initial phase of the anodic pulse. In contrast, the intensity of the 530 cm^−1^ band increased monotonously over the course of the anodic pulse. These changes might represent the formation/crystallization of the Cu_2_O layer terminating the Cu domains. In our previous work, we showed with operando XRD that over the course of an anodic potential pulse, crystalline Cu_2_O domains with a size of up to 3 nm were present at the end of a 10 s anodic pulse^18^. Additionally, operando XAS revealed a minor contribution of other Cu phases (e.g., Cu(II) compounds such as CuO/Cu(OH)_2_) in the initial phase of the electrochemical oxidation, which are likely highly disordered and thus absent in the XRD data. However, they cannot be directly correlated to a specific cationic Cu (surface) species based on SERS.

### Correlations of selectivity, structure, and kinetics

**Fig. 3:**
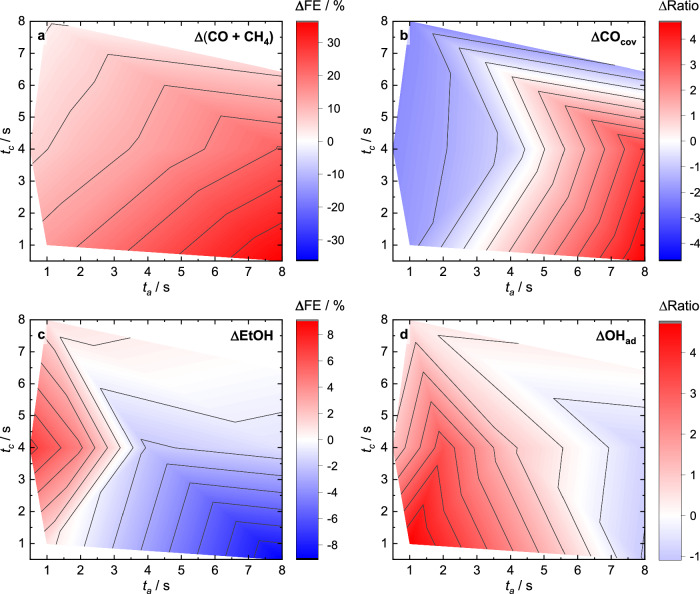
Pulse length-dependent selectivities and adsorbates maps. Changes in product selectivities during pulsed CO_2_RR with *E*_c_ = −1.0 V, *E*_a_ = +0.6 V with respect to the anodic and cathodic pulse lengths after subtraction of the corresponding values under static CO_2_RR conditions at −1.0 V (indicated with Δ). **a** Change (Δ) of the sum of Faradaic efficiencies (ΔFEs) of the main C_1_ products (CO and CH_4_). **b** SERS intensity distribution of ΔCO coverage on Cu. **c** ΔFE of ethanol (EtOH). **d** Normalized SERS intensity distribution of ΔOH_ad_. The selectivity data were taken from identical catalysts included in our previous publication^18^.

To extract the link between the surface adsorbates and the catalytic function, Fig. 3 shows the change in the SERS band intensities (Supplementary Table 2) and in the Faradaic efficiencies (ΔFEs) during pulsed CO_2_RR in comparison to static CO_2_RR conditions at −1.0 V obtained from a variety of anodic and cathodic pulse lengths conditions^18^. We depicted the ΔFEs rather than the partial current densities of the products to compare with the qualitative changes in the relative spectroscopic weight of adsorbate-related bands from SERS. Figure 3b depicts the changes in the CO_cov_ during the cathodic part of pulsed CO_2_RR, which could be determined by the ratio of the CO_s_ and CO_r_ band intensities^33^. The CO surface coverage has been previously shown to be important for the C-C coupling step, and a higher CO coverage could be linked to the increase of C_2+_ products^33^. Under pulsed CO_2_RR, the determined CO coverage is almost unchanged in the C_2_H_4_O_*x*_ regime but decreased in the EtOH regime, while it increased in the C_1_ regime compared to static CO_2_RR conditions (Fig. 3a, b). Thus, the CO coverage does not seem to be the only crucial parameter determining the product selectivity under pulsed conditions.

Therefore, we further investigated the role of OH_ad_, where its beneficial effect was so far only hypothesized or suggested from (theoretical) studies and/or not quantified^22,46,47^. Fig. 3d presents the change of the normalized OH_ad_ intensities during the cathodic pulse (Supplementary Note 2). This map highlights the increased OH_ad_ intensities at shorter anodic pulse lengths (<5 s) in comparison to static CO_2_RR conditions, while the OH_ad_ intensities decreased at longer anodic pulse lengths. This fits to the change of the C_1_ products (Fig. 3a) for lower OH as well as to the enhanced EtOH selectivity at shorter anodic pulse lengths for higher OH intensities (i.e., coverage) (Fig. 3c).

In addition to the average band intensities, the kinetics of the intermediate formation are also expected to significantly influence the observed product generation due to the introduced variations by pulsing the potentials. Therefore, corresponding maps of the ad-/desorption of CO_ad_ and O_ad_, as well as the oxidation and reduction of Cu_2_O depending on the pulse lengths condition, could be created by the use of the time constant of the exponential fits from Fig. 2 (Supplementary Figs. 15–17, Supplementary Tables 3, 4). In the EtOH regime, CO_r_ and CO_s_ vibrations developed faster than they vanished (Supplementary Fig. 15); thus, CO was rapidly available to form EtOH at these pulse lengths. Furthermore, the kinetics of the ad- and desorption of CO_s_ versus CO_r_ (Supplementary Fig. 16), which directly impacts the surface CO coverage, indicate faster kinetics of CO_s_ as compared to CO_r_. This means that the rapidly available surface CO stems first mainly from CO_s_, which has been related to the C–C coupling CO_atop_ in the literature^33^.

Moreover, even though we could not quantify the intensity of bands related to Cu oxides due their strong SERS sensitivity and missing normalization parameter, we can still follow the kinetic evolution of O_ad_ at short anodic pulse lengths and surface Cu_2_O at long anodic pulse lengths (Supplementary Fig. 17). In particular, this demonstrates that Cu-O_ad_ adsorbed kinetically quicker in the EtOH regime compared to the surface Cu_2_O in the C_1_ regime, which could be crucial for the rapid availability of oxygen species to form EtOH.

### Hydroxide to carbon monoxide coverage ratio determines the products

**Fig. 4:**
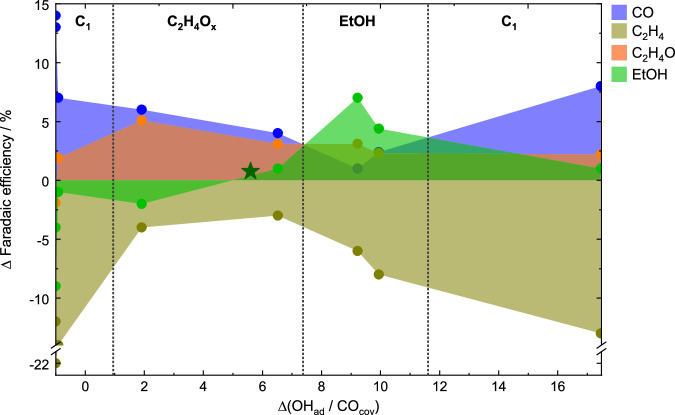
Selectivity change correlated with OH_ad_ / CO_cov_ ratio. Correlations between the selectivity change ΔFE of selected products (CO, ethylene, acetaldehyde, and ethanol) and the Δ (OH_ad_/CO_cov_) under pulsed CO_2_RR conditions after subtracting the corresponding values under static CO_2_RR conditions at −1.0 V. The green star represents the change of the ethanol selectivity during pulsed CO_2_RR up to non-oxidizing potentials at *E*_a_ = 0 V.

To shift the focus from the applied pulse lengths, Fig. 4 directly plots the ΔFEs of selected products such as CO, C_2_H_4_, acetaldehyde and EtOH against the essential adsorbates previously identified, OH_ad_ and CO, which are reflected here by the change of their ratio. The changes were calculated with respect to stationary reaction conditions. The corresponding plots showing all the other products can be found in Supplementary Fig. 18. Figure 4 shows that the FEs of C_2_H_4_ and H_2_ under pulsed CO_2_RR were lower, irrespective of the surface adsorbate composition, while the selectivities toward CO, acetaldehyde, and EtOH were (mostly) increased. In fact, Fig. 4 can be divided into four different product regions depending on the OH_ad_/CO ratio. The lowest OH_ad_/CO ratio (-1, where no OH_ad_ is adsorbed) is characterized by the maximum of C_1_ products, such as CO and CH_4_. Increasing OH_ad_/CO ratios (1–7.3) lead to the increased formation of C_2+_ products such as C_2_H_4_ and acetaldehyde. A further increase of the OH_ad_/CO ratios (7.3–11.6) correlates with the highest ΔFE of EtOH, together with other minor alcohols, while the ΔFEs of CO, C_2_H_4_, and acetaldehyde decreased, highlighting the beneficial effect of enhanced OH coverage for the ethanol formation. Interestingly, the improvement of the CO_2_RR is not only seen in the FE of ethanol but also the partial current density increases at an optimal ratio of co-adsorbed CO and OH during CO_2_RR (Supplementary Fig. 19). However, at even higher ratios of OH_ad_/CO_cov_ (>11.6) the FE of EtOH and ethylene started to decrease, while the FEs of H_2_ and C_1_ products such as CO and COO^-^ (Supplementary Fig. 18a) increased. At these high ratios, the selectivities of EtOH and acetaldehyde were still slightly enhanced as compared to static CO_2_RR conditions^18^.

We note that the decrease of the ethylene FE may be predominantly linked to irreversible (morphological) catalyst changes during the harsh working conditions, as shown in our previous work and will be further discussed later^18^.

In order to verify the dependence of the EtOH selectivity on the OH_ad_ concentration further, pulsed CO_2_RR up to non-oxidizing *E*_*a*_ = 0 V at *t*_c_/*t*_a_ = 4 s/1 s was also measured (Supplementary Fig. 20, Supplementary Table 6). The slight enhancement of EtOH compared to the static conditions goes along with a slight enhancement in OH_ad_ versus CO_cov,_ as also indicated in Fig. 4 (green star), and supports the beneficial effect of OH_ad_ for the EtOH production. These findings highlight the crucial role of OH_ad_ and CO in the formation of EtOH.

## Discussion

**Fig. 5:**
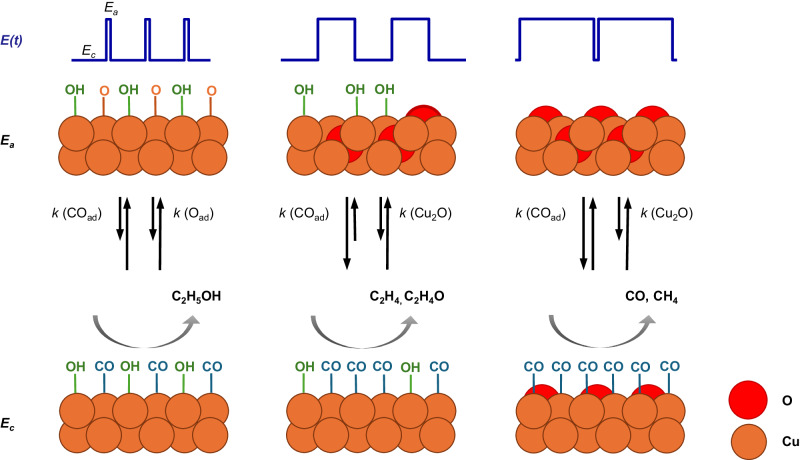
Scheme of adsorbate and selectivity evolution during pulsed CO_2_RR. Depiction of the processes observed during pulsed CO_2_RR with *E*_c_ = −1.0 V, *E*_a_ = +0.6 V of pre-reduced Cu_2_O NCs in dependence of the anodic and cathodic pulse lengths. CO_ad_, OH_ad,_ and O_ad_ coverage are highlighted, and the oxidation state of Cu is indicated, which both contribute to different product selectivities during the cathodic pulse. The rate constant *k* of the CO ad- and desorption, as well as the Cu reduction and oxidation, are schematically specified by the lengths of the arrows to each other, where a longer arrow is correlated to a faster process.

From the results discussed, Fig. 5 suggests the mechanism for pulsed CO_2_RR, where the periodic switching of the applied potential can change the Cu oxidation state and modify the adsorbate coverage of OH and CO with distinct kinetic behavior. Cu oxide-related species only formed during the anodic pulse and rapidly reduced again once the cathodic pulse was applied. At short anodic (*t*_a_ < 2 s) and intermediate cathodic (*t*_c_ = 4 s) pulse lengths, Cu-O_ad_ vibrations and/or CuO_*x*_/(OH)_*y*_ species were detected, which also correlate well with the observation of Cu(II) at short anodic pulses from operando XAS data in our prior study^18^. These CuO_*x*_/(OH)_*y*_ species might be essential for the enhanced EtOH selectivity and are directly oxidized from Cu(0) to Cu(II) during the anodic pulse as no characteristic Cu_2_O bands could be detected. Instead, at longer anodic (*t*_a_ > 2 s) and shorter cathodic pulses (*t*_c_ ≤ 4 s), typical Cu_2_O bands appeared and reflected the growth and crystallization of Cu_2_O over the course of the anodic pulse. Larger amounts of bulk-like Cu_2_O, which were still present at the beginning of the cathodic pulse, led then to an enhancement in the methane selectivity linked to morphology changes, as also seen previously with XAS/XRD^22^.

Most importantly, the Cu redox transitions, the OH surface coverage, and the local pH seem to be intertwined. In general, the concentration of OH^−^ close to and adsorbed on the catalyst surface is expected to increase during pulsed CO_2_RR at −1.0 V. Therein, OH^-^ species are produced by the reduction of H_2_O during the cathodic pulse but are kept close to the catalyst surface due to the positive polarization of the Cu electrode during the anodic pulse. This is expected to increase the local pH during pulsed CO_2_RR compared to static CO_2_RR. The limited time at short *t*_a_ values prevents the formation of an ordered Cu_2_O phase and favors the formation of the detected Cu–O_ad_ and/or CuO_*x*_/(OH)_*y*_ species. At longer oxidizing pulses, the low OH coverage values likely result from OH_ad_/OH^−^ consumption for the Cu_2_O formation (2 Cu + 2 OH^−^ → 2 Cu–OH → Cu_2_O + H_2_O)^22,24^, leading to a decrease in the local pH during CO_2_RR, which is known to favor methane and CO production^22^. However, too high coverages of OH_ad_ start to block CO adsorption sites and prevent C–C coupling, which leads to an increase in C_1_ products and HER^48^.

It is important to note that the decrease of the ethylene and increase of the methane FE is linked to irreversible (morphological) catalyst changes caused by the pulsed reaction conditions (see ex-situ SEM images in Supplementary Fig. 21), which are especially prominent at longer anodic pulse length durations as also shown in our previous work^18^. Here, qualitative agreement to very high roughness and/or Cu dissolution of the Cu NCs could also lead to an increased population of low-coordinated sites, which are selective for hydrogen^49^. For example, sub-nanometer Cu clusters, likely formed under these continuous harsh redox cycles, were observed to be selective for methane formation^50,51^. This explains that under pulsed conditions, the highest CO coverage (without co-adsorbed OH_ad_) was identified to enhance methane selectivities, while under less harsh stationary conditions, the highest CO coverage (usually with co-adsorbed OH_ad_) was attributed to the highest C_2+_ product yield^6,33^. This suggests that under these pulsed reaction conditions, the C-C coupling is significantly hampered, leading to the reduction of the adsorbed CO to methane. To facilitate EtOH formation, the CO and OH coverage have to be balanced during pulsed CO_2_RR to enhance the protonation of the *CH_*x*_CO intermediates and/or hamper its O removal^52^, e.g., by a sufficient OH coverage blocking adjacent adsorption sites. Lower OH or higher CO coverages and, thus, more similar conditions as under static CO_2_RR shift the selectivity toward protonated C_2+_ products such as ethylene and acetaldehyde. Therefore, we hypothesize that increasing the interfacial availability of base (OH) sites under (acidic) CO_2_RR conditions might lead to even higher EtOH formation.

In the EtOH regime, we found faster kinetics of CO adsorbate formation, which are expected to facilitate CO–CO dimerization and, thus, decrease C_1_ product formation. The fast kinetics of the CO coverage in the EtOH regime might also be beneficial for the fast stabilization of OH_ad_ since CO_ad_ has already been demonstrated to stabilize OH_ad_ by DFT calculations^46^. Furthermore, the fast kinetics of Cu–O_ad_ in the EtOH regime compared to the formation of surface Cu_2_O in the C_1_ regime during the anodic pulse demonstrate the rapid availability of oxygen species with short anodic pulse durations that are needed for ethanol formation.

In conclusion, this study revealed the link between the changes in the adsorbate structure and composition and the catalytic function of oxide-derived Cu nanocatalysts during pulsed CO_2_RR by utilizing time-resolved operando SERS. By the implementation of sub-second time resolution, the development of characteristic adsorbates such as OH and CO could be tracked during each individual pulse. In this way, the observed ethanol enhancement could be attributed to an optimal OH and CO surface coverage, which was found to be selectivity-determining towards alcohols over hydrocarbons. Furthermore, these OH species had a significantly lower concentration during stationary CO_2_RR and were not present in noticeable amounts on Cu_2_O-derived working catalysts. Thus, only the intermittent formation of an OH/O-covered Cu surface triggers the continuous regeneration of the OH/CO-covered Cu catalyst during CO_2_RR. Furthermore, the preferable CO ad-/desorption kinetics were found to contribute to higher ethanol yields. On the contrary, OH_ad_ was found to be converted into bulk-like Cu_2_O species, which also led to the decrease of the near-surface pH and the formation of unfavorable C_1_ products, such as methane and CO, as well as tremendously different reduction mechanisms, not being able to stabilize the OH species. All in all, this study underlines the urgency of time-resolved surface-sensitive techniques such as operando SERS to understand the reaction mechanism of pulsed CO_2_RR in order to favor the desired product selectivities. Furthermore, we finally confirmed experimentally the importance of surface base (OH) sites within the CO_2_RR mechanistic framework which will pave the road for in-depth investigations and novel catalyst design approaches.

## Methods

### Catalyst preparation

Cu_2_O NCs were synthesized by a ligand-free method, as described in our previous study^6^. The reagents were purchased from Sigma Aldrich in ACS grade and used without further purification. 5 mL of a CuCl_2_*2 H_2_O solution (0.1 M) and 15 mL of a NaOH solution (0.2 M) were added to 200 mL of ultrapure water (>18 MΩ cm) at room temperature, and the solution was stirred for 5 min. Then, 10 mL of an l-ascorbic acid solution (0.1 M) was added to the mixture, and the solution was further stirred for 1 h. The solution was centrifuged and washed three times, twice with an ethanol-water mixture (1:1) and once with pure ethanol. The product was dried in a vacuum overnight, and the obtained powder was stored in the glove box.

To prepare the electrodes, 1 mg of the catalyst powder was dispersed in 0.5 mL of pure ethanol and ultrasonicated for 15 min to reach a concentration of Cu_2_O of 2 mg mL^−1^. For the operando Raman measurements, 31 µL of the dispersion were drop-casted on one side of a polished glassy carbon electrode (8 × 8 mm, Glassy Carbon SIGRADUR®, HTW) and dried at 60 °C for 5 min to obtain a Cu_2_O mass-loading of ~100 µg cm^−2^.

### Electrolyte preparation

0.1 M KHCO_3_ (Alfa Aesar, 99.7–100.5%) was purified with a cation-exchange resin (Chelex 100 Resin, Bio-Rad) and saturated with CO_2_ (99.995%) for at least 15 min until a pH of 6.8 was reached.

### Operando surface-enhanced Raman spectroscopy

Operando SERS was performed with a Raman spectrometer (Renishaw, InVia Reflex) coupled with an optical microscope (Leica Microsystems, DM2500M) together with a motorized stage for sample tracking (Renishaw, MS300 encoded stage). Calibration of the system was carried out by using a Si(100) wafer (520.5 cm^-1^). A near-infrared laser (Renishaw, RL785, *λ* = 785 nm, *P*_max_ = 500 mW, grating 1200 and 1800 lines mm^−1^), as well as a HeNe laser (Renishaw, RL633, *λ* = 633 nm, *P*_max_ = 17 mW, grating 1800 lines mm^−1^), were used as excitation sources. The backscattered light was Rayleigh-filtered and directed to a CCD detector (Renishaw, Centrus). For the operando measurements, the excitation source was focused on the surface of the sample, and Raman scattering signals were collected with a water immersion objective (Leica microsystems, ×63, numerical aperture of 0.9). The objective was protected from the electrolyte by a Teflon (FEP) film (Goodfellow, film thickness of 0.0125 mm), which was wrapped around the objective.

The electrochemical measurements were conducted at room temperature in a home-built spectro-electrochemical flow cell made of PEEK and controlled by a Biologic SP240 potentiostat (Supplementary Fig. 1). The cell was equipped with a leak-free Ag/AgCl reference electrode (LF-1-63, 1 mm OD, Innovative Instruments, Inc.) positioned close to the sample and a Pt counter electrode in the outlet of the flow. The working electrode with the catalyst drop-casted on glassy carbon was mounted from the bottom of the cell, and the area of the exposed catalyst was 0.25 mm^2^. The electrolyte (0.1 M KHCO_3_) was CO_2_-saturated (pH = 6.8) in its reservoir (*V* = 50 mL) outside of the Raman system and, from there, pumped through the cell with a peristaltic pump (PLP 380, Behr Labor-Technik).

The potentials in this manuscript were all converted to the RHE scale (${E}({{{{{{\rm{vs.}}}}}}\; {{{{{\rm{RHE}}}}}}})={E}({{{{{{\rm{vs.}}}}}}\; {{{{{\rm{Ag}}}}}}}/{{{{{\rm{AgCl}}}}}})+0.242\,{{{{{\rm{V}}}}}}+0.059\,{{{{{\rm{V}}}}}}\times {{{{{{\rm{pH}}}}}}-{{{{{{\rm{iR}}}}}}}}$) and corrected for *iR* drop as determined by electrochemical impedance spectroscopy.

The collection time of each spectrum depends on the applied electrochemical protocol. For the pulsed CO_2_RR experiments at the cathodic potential *E*_c_ = −1.0 V and the anodic potential *E*_a_ = +0.6 V, acquisition times between 0.1 and 0.8 s were used, depending on the cathodic and anodic pulse lengths *t*_c_ and *t*_a_, respectively, to obtain at least three data points per pulse. The exact temporal resolutions for the low Raman shift region are given in Supplementary Table 1. To obtain a high time resolution, usually (if not stated differently), the static Raman mode in the region of 55–1272 cm^−1^ was applied together with the 785 nm laser and the 1200 lines mm^−1^ grating. In the region of 1700–2600 cm^−1^, the 633 nm laser and the 1800 lines mm^−1^ grating were used. The Raman data were first processed using the Renishaw WiRE 5.2 software to normalize the data and remove cosmic rays. Octave® scripts were written to combine the Raman and the electrochemical data, to fit characteristic Raman bands, and to average the Raman spectra. Averaged Raman spectra were obtained by averaging the Raman data points collected at the same times after the onset of each pulse cycle.

### Ex situ scanning electron microscopy

Ex situ SEM images of the samples prior to and after the different electrocatalytic conditions were measured using an SEM (Apreo SEM, Thermo Fisher Scientific) with an in-lens secondary electron detector. The samples were deposited on glassy carbon and directly rinsed with ultrapure water (>18 MΩ cm) after each electrocatalytic CO_2_RR measurement to avoid electrolyte salt contamination on the sample.

### Selectivity measurements

The main part of the selectivity measurements was already published in our previous work^18^ except for the new measurements acquired at *E*_c_ = −1.0 V, *E*_a_ = 0 V (versus RHE and *iR* drop corrected) and *t*_c_ = 4 s, and *t*_a_ = 1 s for a total duration of 4000 s. The measurements were carried out in an H-type cell equipped with an anion-exchange membrane (Selemion AMV, AGC) separating the cathodic and the anodic compartments and controlled by an Autolab (Metrohm) potentiostat. A leak-free Ag/AgCl reference electrode (LF-1, Alvatek) served as the reference electrode, a platinum gauze electrode (MaTecK, 3600 mesh cm^−2^) as the counter electrode, and Cu_2_O NCs deposited on carbon paper as the working electrode. The electrolyte was CO_2_-saturated 0.1 M KHCO_3_. Gas products were detected and quantified every 15 min by online gas chromatography (GC, Agilent 7890B), equipped with a thermal conductivity detector and a flame ionization detector. Liquid products were analyzed after each measurement with a high-performance liquid chromatograph (Shimadzu Prominence), equipped with a NUCLEOGEL SUGAR 810 column and a refractive index detector, and a liquid GC (Shimadzu 2010 plus), equipped with a fused silica capillary column and a flame ionization detector. The Faradaic efficiencies were calculated by taking only the cathodic part into account since there is no catalytic activity during the anodic pulse. Specifically, for the calculations of the Faradaic efficiencies of the gas products, the cathodic current was weighted by the term $\frac{{t}_{c}}{{t}_{c}+{t}_{a}}$ to correct for effective time under CO_2_RR, while for the Faradaic efficiencies of the liquid products, just the cathodic charge was taken into account.

### Supplementary information

Supplementary Information

Peer Review File

## Data Availability

Additional SERS data, fitting parameter, time constants and additional CO_2_RR data are given in the Supplementary Information. The raw SERS data (which require specialized software to process) are available from the corresponding authors on request.
