# Supplementary material for: Operando Raman spectroscopy uncovers hydroxide and CO species enhance ethanol selectivity during pulsed CO2 electroreduction
Source: Nat Commun. 2024 May 11;15:3986. doi: 10.1038/s41467-024-48052-3 (PMC11088695; doi:10.1038/s41467-024-48052-3)
Supplement: Supplementary file 1 — Supplementary Information [file 41467_2024_48052_MOESM1_ESM.pdf]

Supplementary Information

Operando Raman Spectroscopy Uncovers Hydroxide and CO Species Enhance Ethanol Selectivity during Pulsed CO_2 Electroreduction

Antonia Herzog[1,2], Mauricio Lopez Luna[1,3], Hyo Sang Jeon[1,4], Clara Rettenmaier[1], Philipp Grosse[1], Arno Bergmann[1], Beatriz Roldan Cuenya[1]**

[1]Department of Interface Science, Fritz-Haber Institute of the Max-Planck Society, 14195 Berlin, Germany

[2]Current address: Massachusetts Institute of Technology, Research Laboratory of Electronics, 77 Massachusetts Ave, Cambridge, MA 02139, USA

[3]Current address: Chemical Sciences Division, Lawrence Berkeley National Laboratory, Berkeley, CA 94720, USA

[4]Current address: Korea Institute of Science and Technology, 5 Hwarang-ro 14-gil, Wolgok 2(i)-dong, Seongbuk-gu, Seoul, South Korea

*abergmann@fhi-berlin.mpg.de, roldan@fhi-berlin.mpg.de

Supplementary Notes

Supplementary Note 1.

Procedure of catalyst pre-reduction followed by pulsed CO_2 electroreduction (CO_2RR)

In a first step, Cu_2O NCs were pre-reduced in an electrochemical operando Raman flow cell setup (Supplementary Fig. 1) in 0.1 M CO_2-saturated potassium bicarbonate ($KHCO_3$) by performing a linear sweep with 10 mV s^{-1} from open circuit potential (OCP) to -1.0 V (vs RHE, for all shown potentials in this study), where the potential was kept for 15 min. Subsequently, pulsed CO_2RR was applied. Both steps were tracked by operando SERS at the same time, and different spectral regions of interest are highlighted in Supplementary Fig. 2.

Supplementary Note 2.

Details of SERS band normalization

SERS band intensities can vary strongly depending on the SERS enhacement of our employed Cu-based cataylst. Here, the SERS enhacement alters under different pulsed CO_2RR conditions due to morphological and compositional changes. Thus, for quantification SERS band intensity normalization is crucial. The CO coverage can be determinded by the CO_s and CO_r intensity ratio of the same spectrum and is therefore independend of the absolute intensities, as shown in our previous study.[1] To further quantify the relative amount of hydroxide, we normalized the band by the CO_s and CO_r band of the same spectrum.

Supplementary Figures

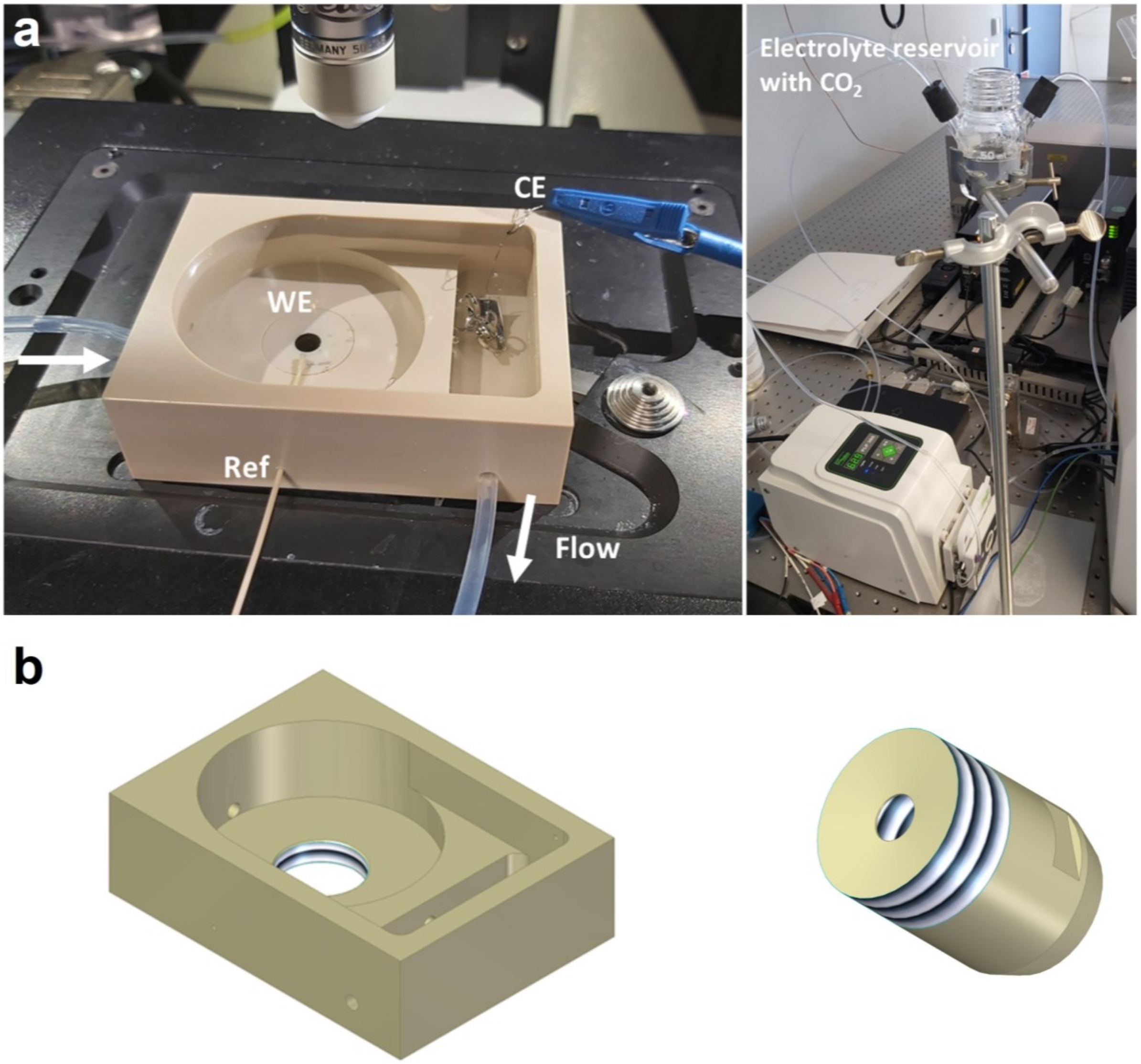

Supplementary Figure 1. Pictures of our home-built spectro-electrochemical operando Raman flow cell setup (a) and schemes of the cell body ((b), left) and sample holder, where the sample is placed below the hole ((b), bottom right).

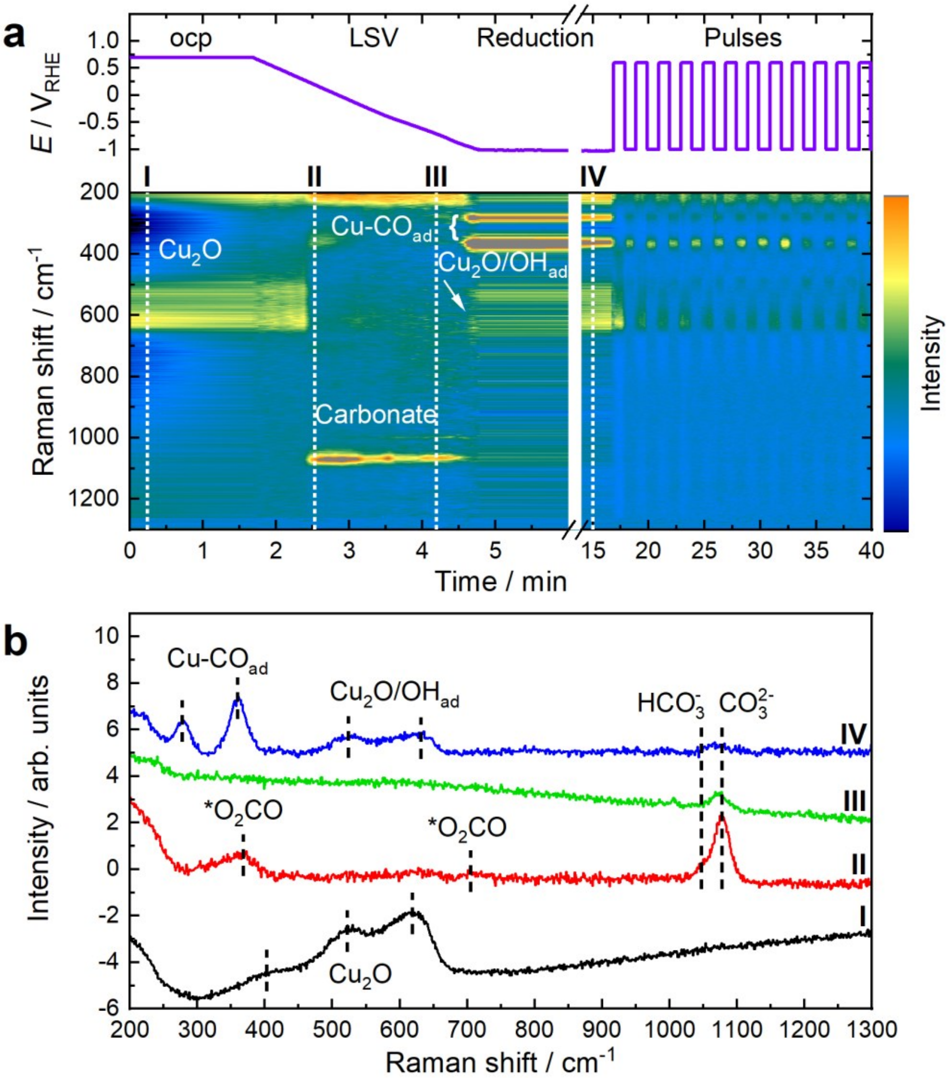

Supplementary Figure 2. Experimental protocol during a representative pulsed experiment. (a) Applied potential over time during open circuit potential (ocp), linear sweep voltammetry (LSV), as well as reduction and pulses (top) together with the temporal evolution of the SERS signal intensity of Cu_2O nanocubes (NCs) in the range of 200-1300 cm^{-1} with highlighted characteristic bands. (b) Selected normalized SERS spectra from (a) with highlighted Raman shifts, which correspond in (I) to Cu_2O species (402, 526, 620 cm^{-1}); in (II) and (III) to adsorbed bidentate carbonate species ($^*O_2CO$, 360, 705 cm^{-1}), to carbonate (CO_3^{2-}, 1074 cm^{-1}) and to bicarbonate (HCO_3^-, 1050 cm^{-1}); and in (IV) to the Cu-CO rotation (280 cm^{-1}) and Cu-CO stretching bands (360 cm^{-1}) and to Cu_2O and/or OH_{ad} species (530, 630 cm^{-1}).[1-4]

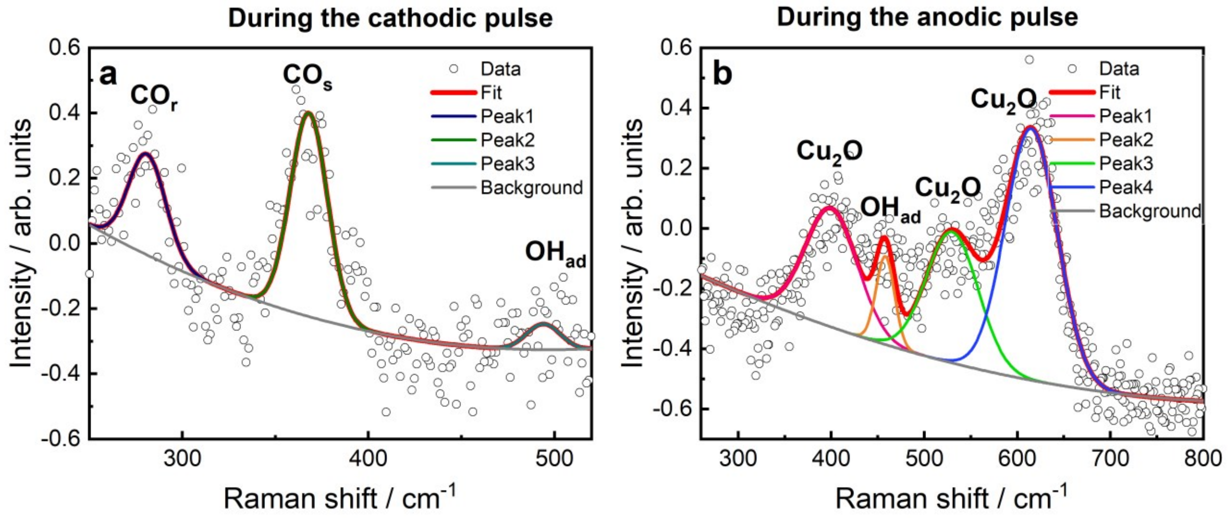

Supplementary Figure 3. Exemplary fits of characteristic peaks of the normalized SERS spectra during the cathodic and anodic pulses of CO_2RR for $t_c = t_a = 4$ s and $E_c = -1.0$ V and $E_a = +0.6$ V.

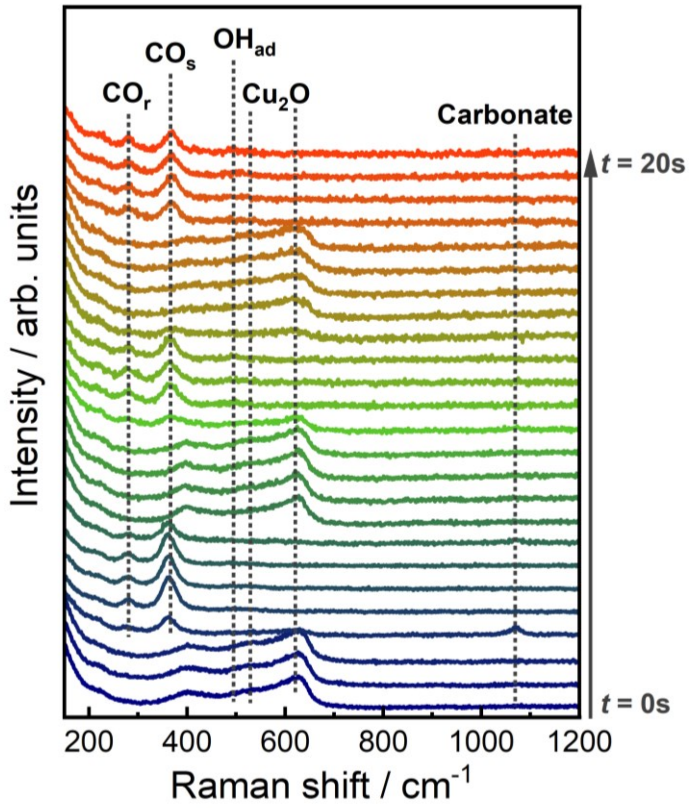

Supplementary Figure 4. Normalized SERS spectra during the first 20 s of pulsed CO_2RR at $t_c = t_a = 4$ s with $E_c = -1.0$ V and $E_a = +0.6$ V. Characteristic bands are highlighted.

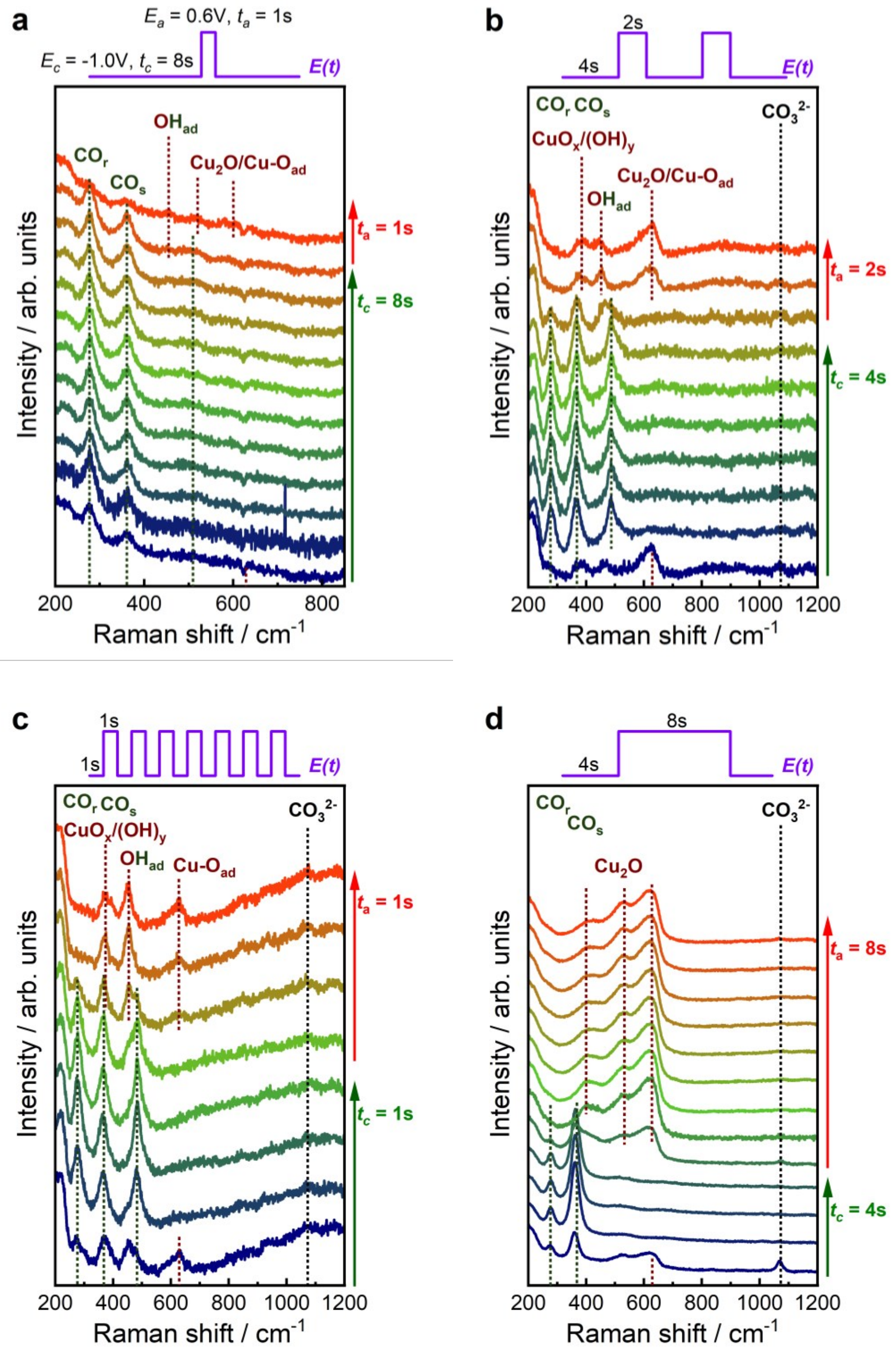

Supplementary Figure 5. Normalized SERS spectra of pulsed potential CO_2RR measurements, where the green arrow indicates the cathodic part at E_c = -1.0 V and the red arrow the anodic part at E_a = +0.6 V with the pulse lengths of (a) t_c = 8 s, t_a = 1 s, (b) t_c = 4 s, t_a = 2 s, (c) t_c = t_a = 1 s and (d) t_c = 4 s, t_a = 8 s averaged over one pulse sequence. Characteristic SERS bands are highlighted. (a) shows at 622 cm^{-1} a negative artefact resulting from the grading in this measurments.

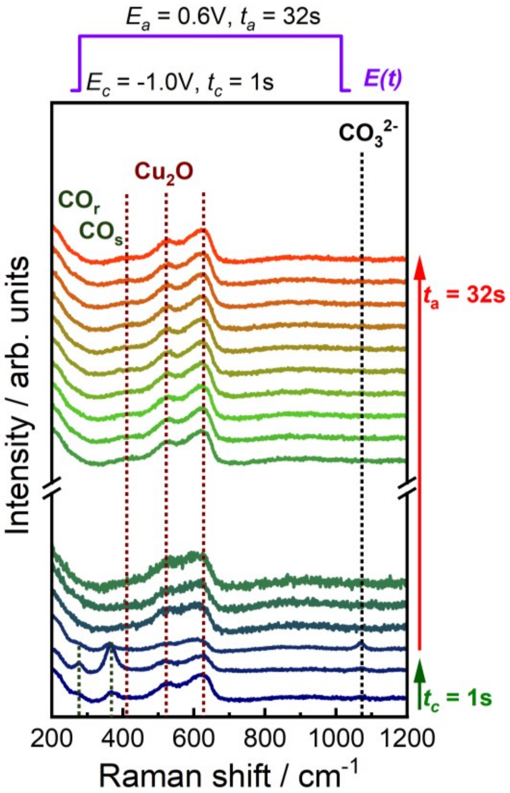

Supplementary Figure 6. Normalized SERS spectra of pulsed potential CO_2RR measurements, where the green arrow indicates the cathodic part at E_c = -1.0 V and the red arrow the anodic part at E_a = +0.6 V with the pulse lengths of t_c = 1 s, t_a = 32 s averaged over one pulse sequence. Characteristic SERS bands are highlighted.

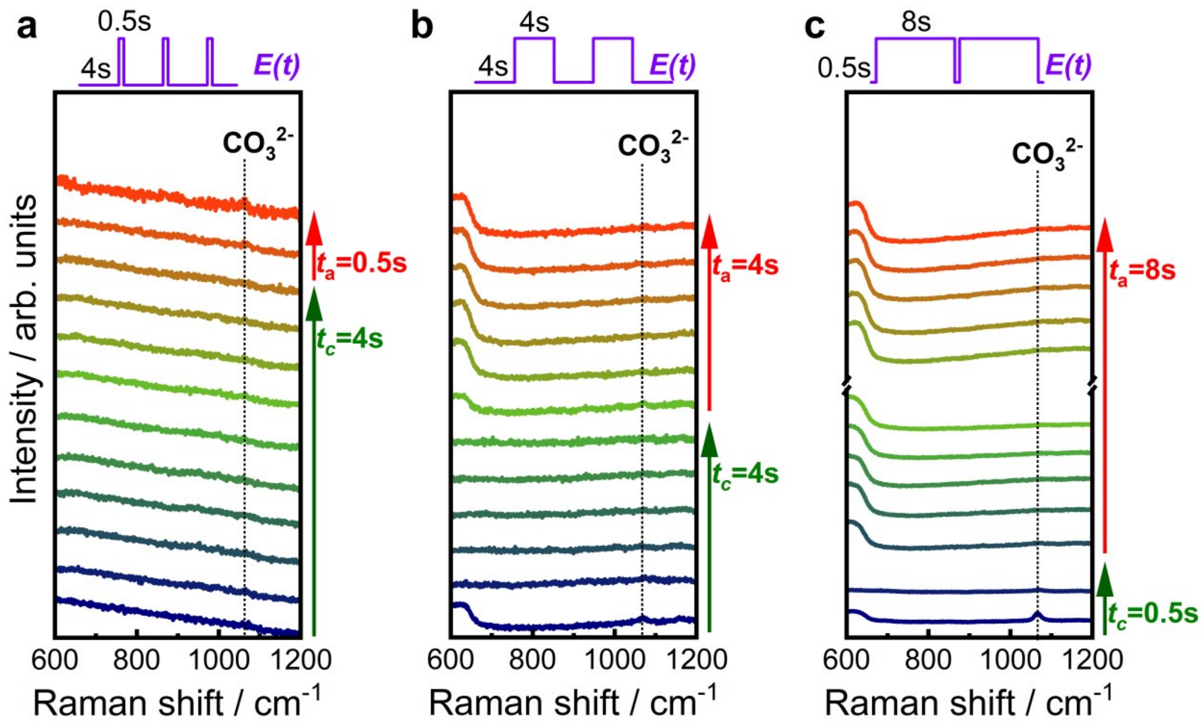

Supplementary Figure 7. Normalized SERS spectra of the carbonate region from 600-1200 cm^{-1} of pulsed potential CO_2RR measurements where the green arrow indicates the cathodic part at E_c = -1.0 V and the red arrow the anodic part at E_a = +0.6 V with the pulse lengths of (a) t_c = 4 s, t_a = 0.5 s, (b) t_c = t_a = 4 s and (c) t_c = 0.5 s, t_a = 8 s averaged over one pulse sequence. Characteristic SERS bands are highlighted.

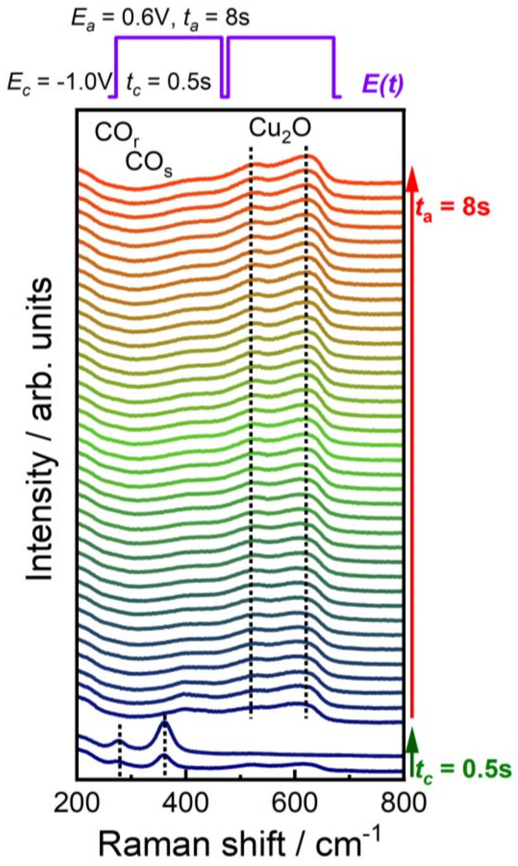

Supplementary Figure 8. Complete set of normalized SERS spectra of pulsed potential CO_2RR measurements where the green arrow indicates the cathodic part at E_c = -1.0 V and the red arrow the anodic part at E_a = +0.6 V with the pulse lengths of t_c = 0.5 s, t_a = 8 s averaged over one pulse sequence. Characteristic SERS bands are highlighted.

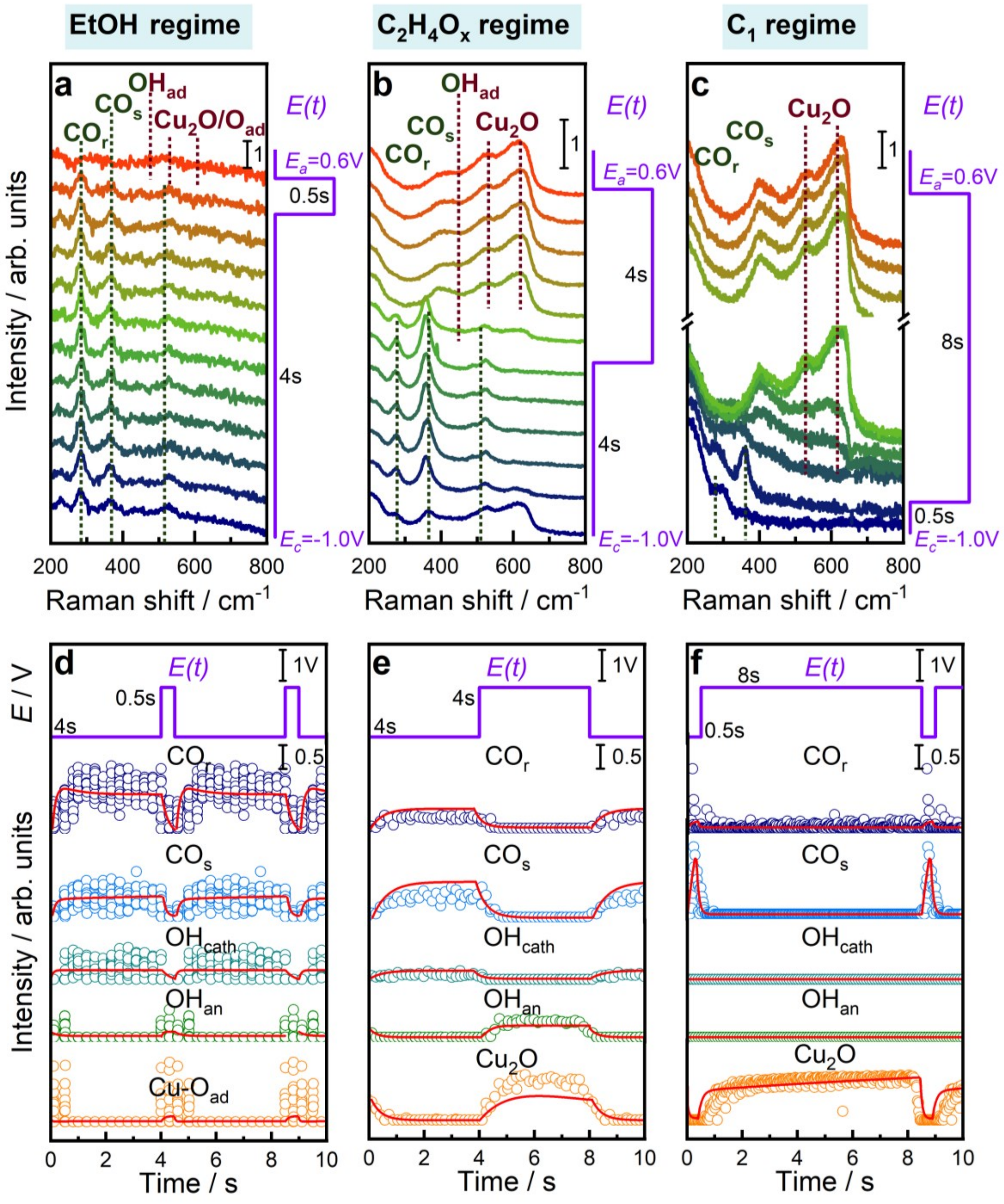

Supplementary Figure 9. (a-c) Normalized SERS spectra on freshly individually prepared electrodes analogue to those in Fig. 2 but carried out on a different sample during pulsed CO_2RR with varying pulse lengths at E_c = -1.0 V and E_a = +0.6 V and (d-f) intensities of the fits of characteristic SERS bands averaged over one pulse sequence at selected pulse lengths. The red lines denote the exponential fits from Fig. 2 and serve as comparison. Despite some small differences arising from changes in the surface structure when comparing differently freshly prepared samples, these reproducibility measurements reported here still align very well with the results in Fig. 2.

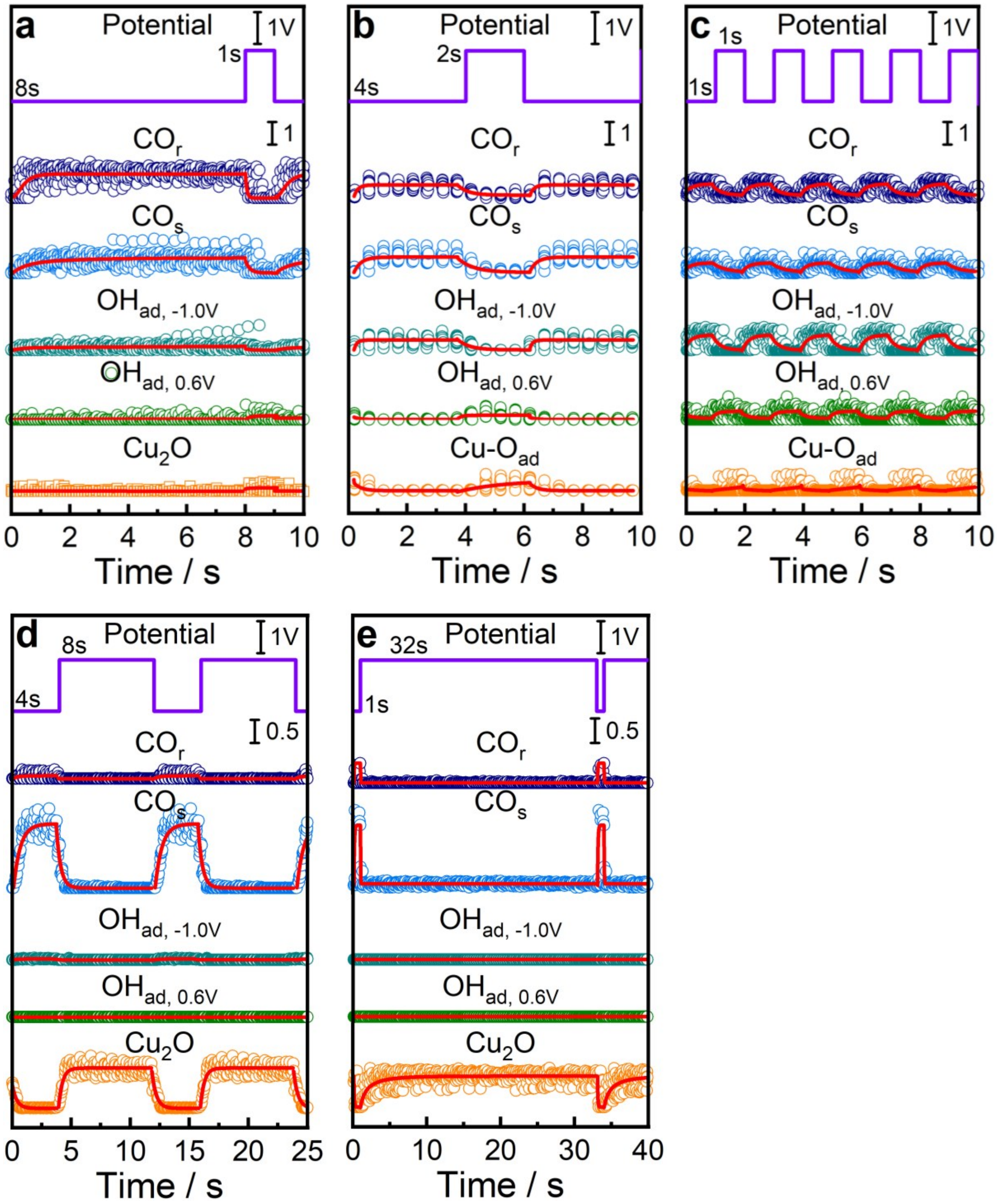

Supplementary Figure 10. Applied potential and intensities of fits of characteristic SERS bands of pulsed potential CO_2RR at E_c = -1.0 V and E_a = +0.6 V with different pulse lengths averaged over one pulse sequence (a-e). The data points are the intensity fits of the Cu-CO rotation (CO_r at 280 cm^{-1}, dark blue), the Cu-CO stretching (CO_s at 360 cm^{-1}, light blue), Cu-OH_{ad} (OH_{ad} at 495 cm^{-1} at -1.0 V in turquoise and 450 cm^{-1} at +0.6 V in green), Cu_2O (sum of 530 and 620 cm^{-1} divided by two, orange) and Cu-O_{ad} (610 cm^{-1}, orange) bands. The red lines represent the exponential fit and are guides for the eyes.

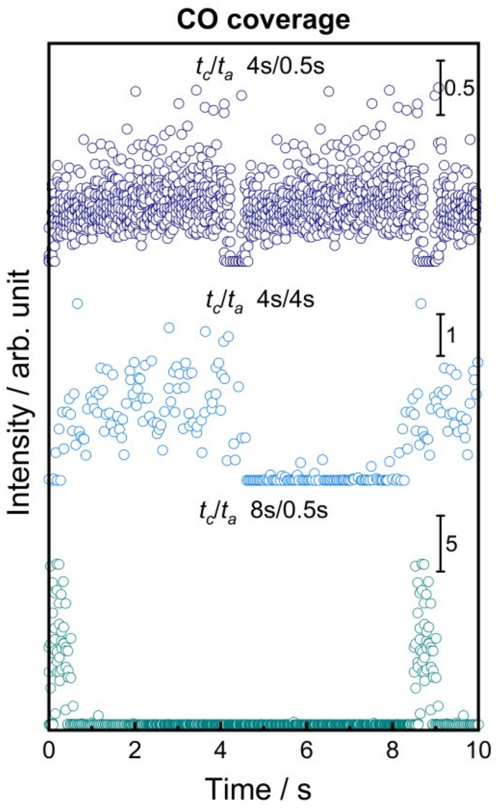

Supplementary Figure 11. CO coverage of selected pulse lengths over one averaged pulse sequence of pulsed potential CO_2RR measurements with E_c = -1.0 V, E_a = +0.6 V. The CO coverage was determined by the ratio of the intensity of CO_s and CO_r as demonstarted in our previous study.[1]

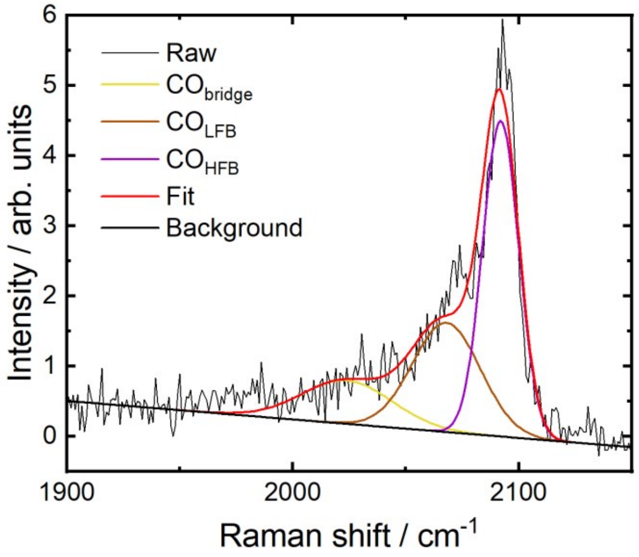

Supplementary Figure 12. Exemplary fits of the C-O vibration region of a normalized SERS spectrum with the contribution of bridge CO (CO_{bridge} at 2030 cm^{-1}), low-frequency band linear CO (CO_{LFB} at 2065 cm^{-1}), and high-frequency band linear CO (CO_{HFB} at 2095 cm^{-1}) during pulsed CO_2RR with the cathodic pulse at t_c = 4 s, t_a = 8 s with E_c = -1.0 V and E_a = +0.6 V.

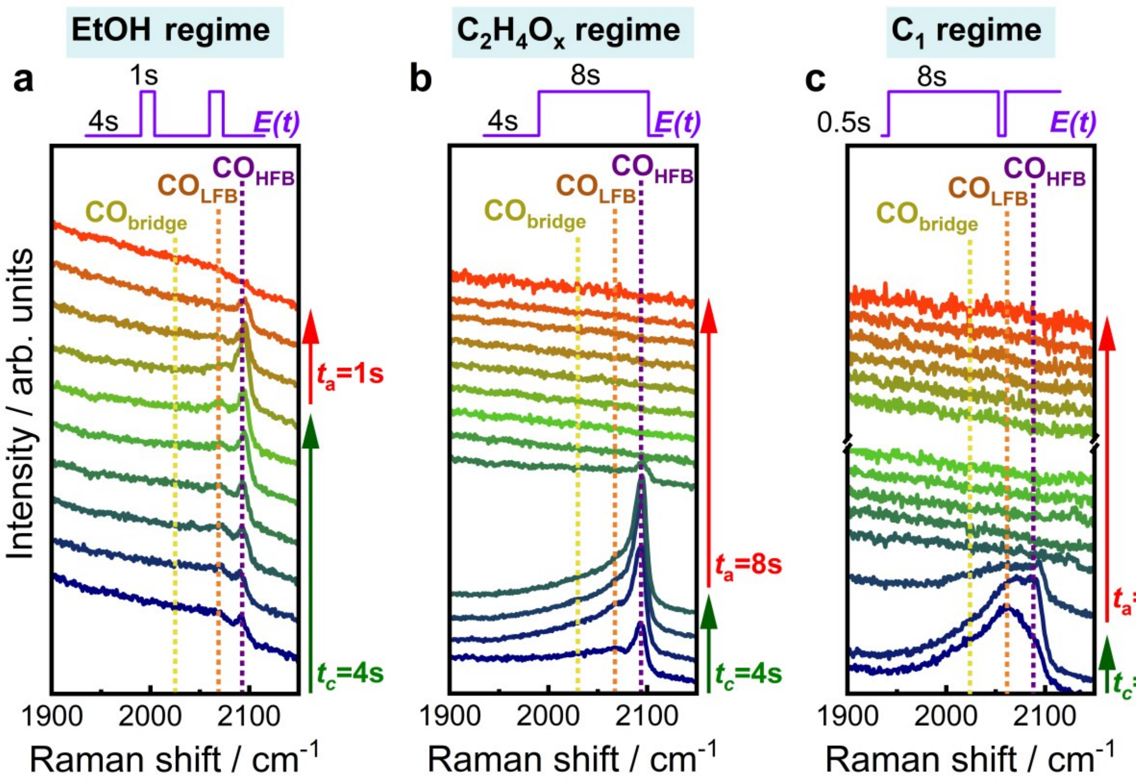

Supplementary Figure 13. Normalized SERS spectra of the C-O vibration region from 1900-2150 cm^{-1} of pulsed potential CO_2RR measurements with varied pulse lengths during E_c = -1.0 V and E_a = +0.6 V averaged over one pulse sequence from bottom to top with green arrow showing the cathodic part and red arrow the anodic part. The pulse lengths are in (a) t_c = 4 s, t_a = 1 s (ethanol regime), (b) t_c = 4 s, t_a = 8 s (ethylene/acetaldehyde regime), and (c) t_c = 0.5 s, t_a = 8 s (C_1 regime). The different C-O configurations such as CO_{bridge}, linear low-frequency CO (CO_{LFB}), and high-frequency CO (CO_{HFB}) are highlighted. Individual spectra were collected with an acquisition time of 0.25 spectra/s.

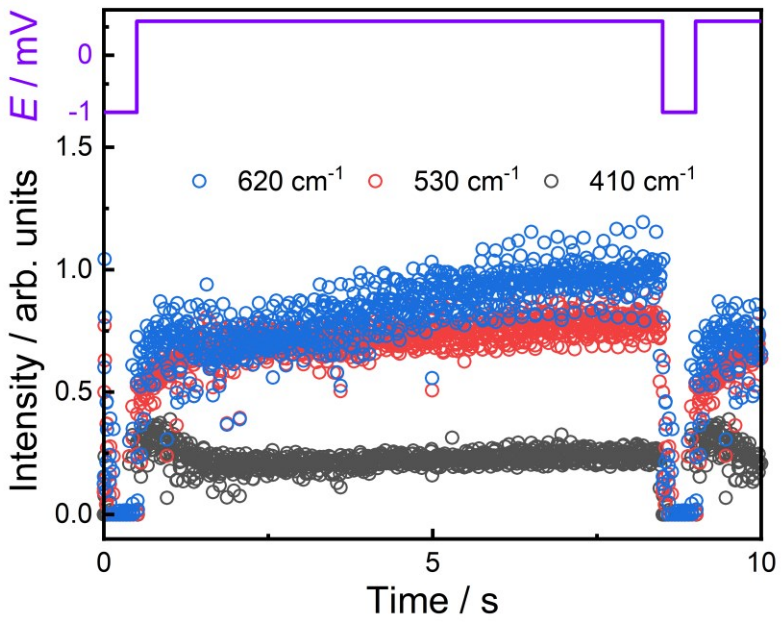

Supplementary Figure 14. Applied potential and intensities of fits of Cu_2O bands at 410, 530, and 620 cm^{-1} during pulsed potential CO_2RR at E_c = -1.0 V and E_a = +0.6 V at t_c = 0.5 s and t_a = 8 s (C_1 regime) averaged over one pulse sequence.

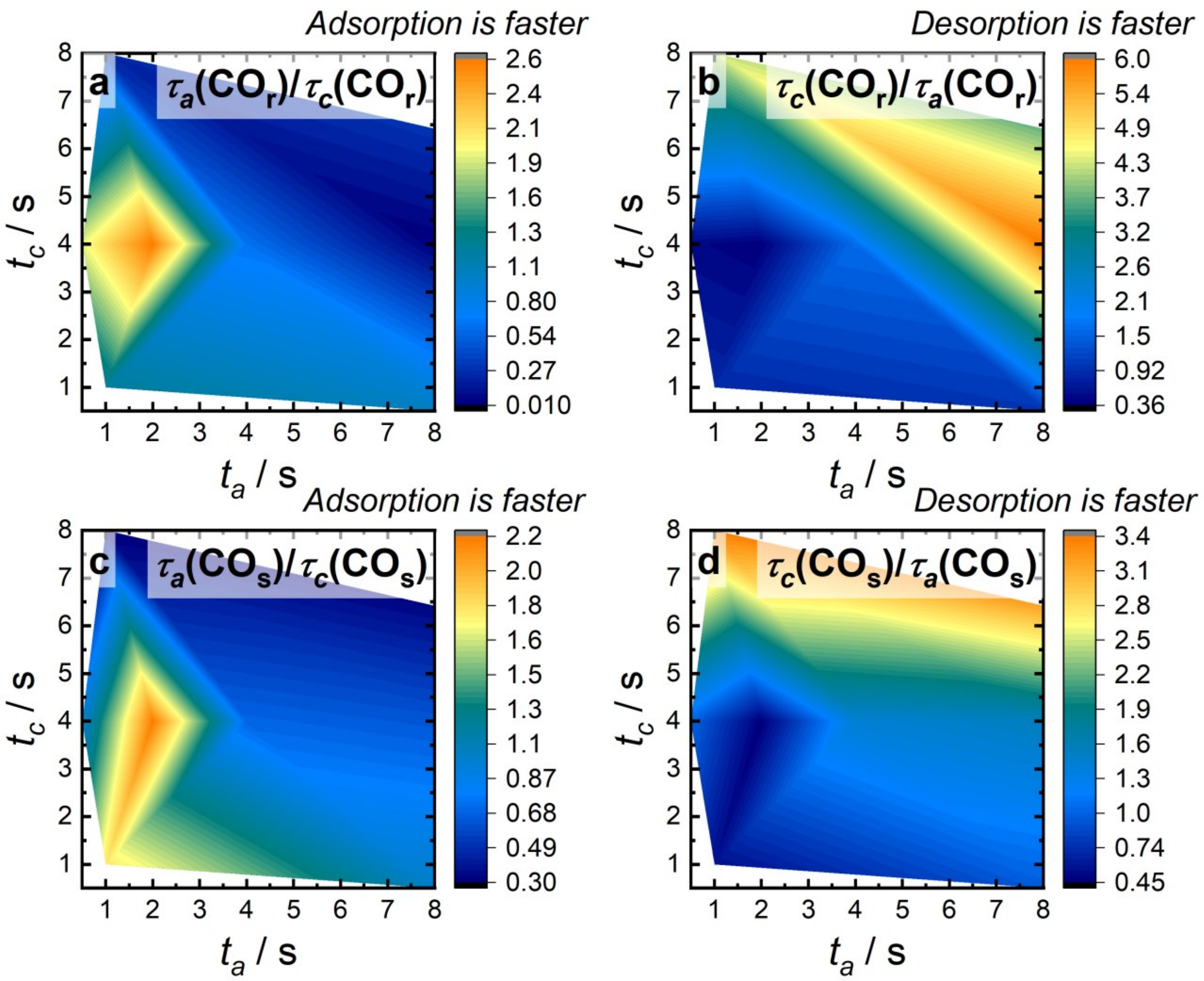

Supplementary Figure 15. Ratios of time constants τ during ad- and desorption of CO_r (a-b) and CO_s (c-d) in dependence of the pulse lengths of pulsed potential CO_2RR measurements with E_c = -1.0 V, E_a = +0.6 V.

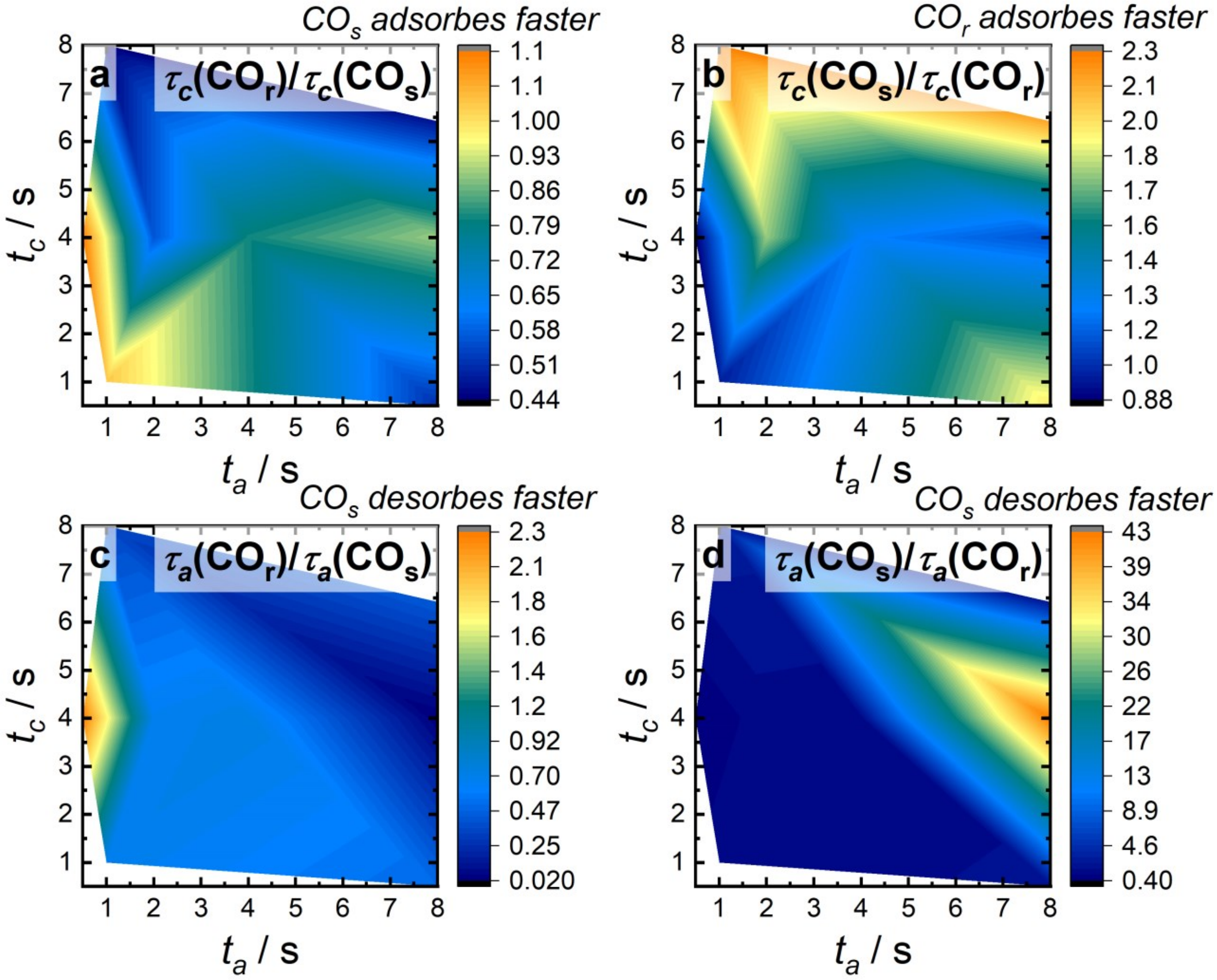

Supplementary Figure 16. Ratio of time constants τ during the adsorption of (a) CO_r versus CO_s and of (b) CO_s versus CO_r during the cathodic pulse, as well as the ratio during desorption of (c) CO_r versus CO_s and of (d) CO_s versus CO_r during the anodic pulse in dependence of the pulse lengths of pulsed potential CO_2RR measurements with E_c = -1.0 V, E_a = +0.6 V.

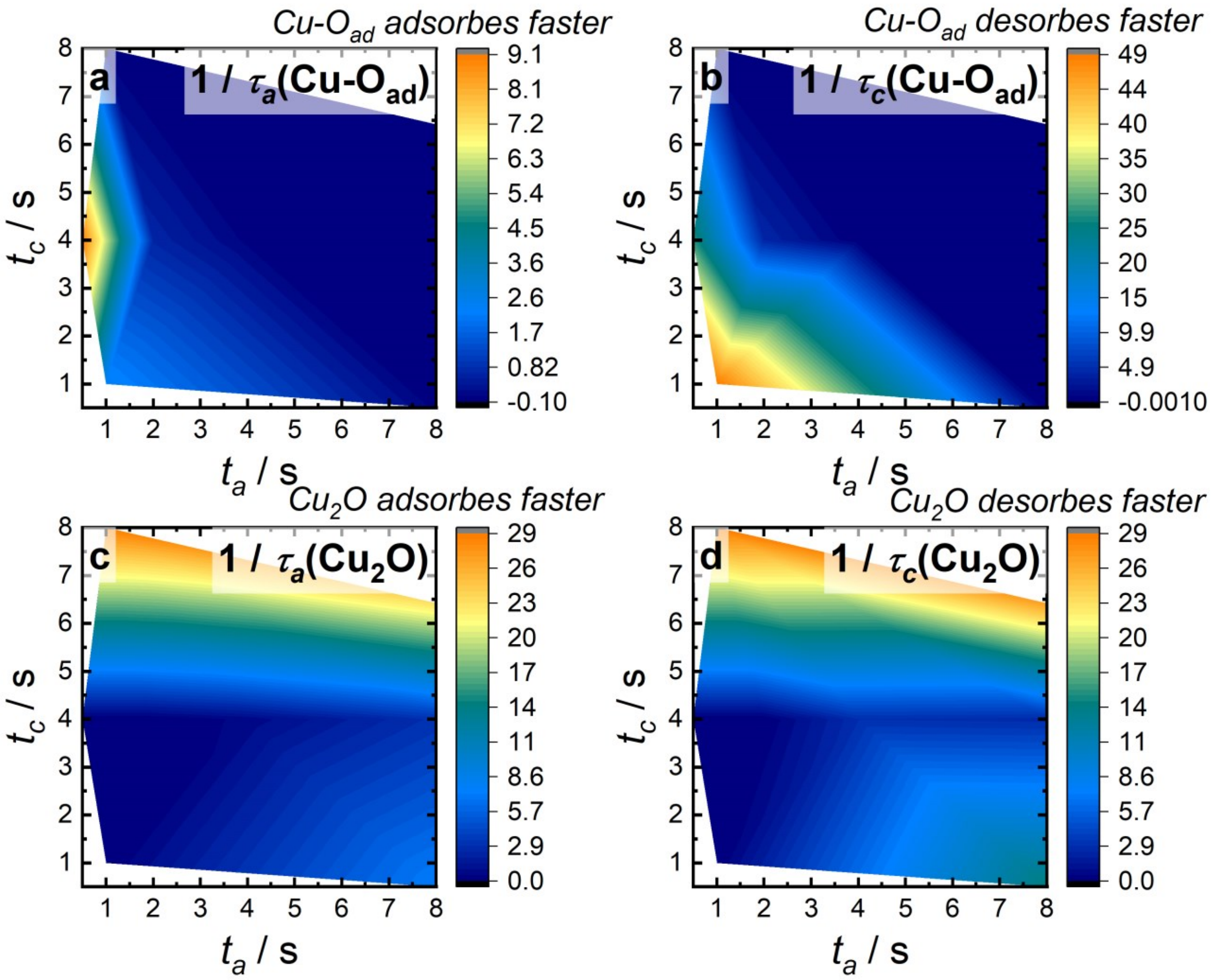

Supplementary Figure 17. Ratios of time constants τ during the adsorption of Cu-O_{ad} (a) and the oxidation of Cu to Cu_2O (c) at the anodic pulse and during the desorption of Cu-O_{ad} (b) and the reduction of Cu_2O to Cu (d) at the cathodic pulse in dependence of the pulse lengths of pulsed potential CO_2RR measurements with E_c = -1.0 V, E_a = +0.6 V.

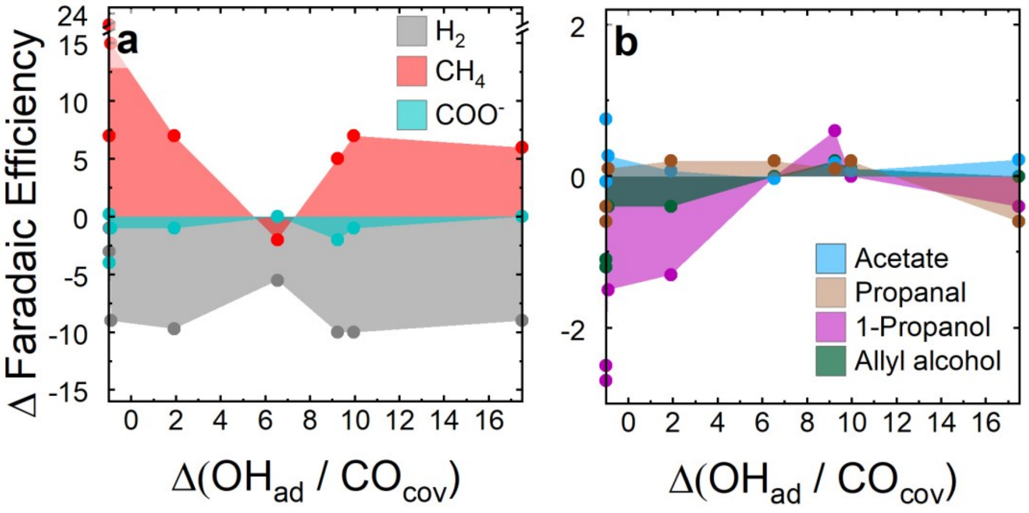

Supplementary Figure 18. Correlations between the selectivity change ΔFE of selected C_1 (a) and minor products (b) and Δ(OH_{ad} versus CO_{cov}) in arb. units under pulsed CO_2RR conditions after subtraction of the corresponding values under static CO_2RR conditions at -1.0 V.

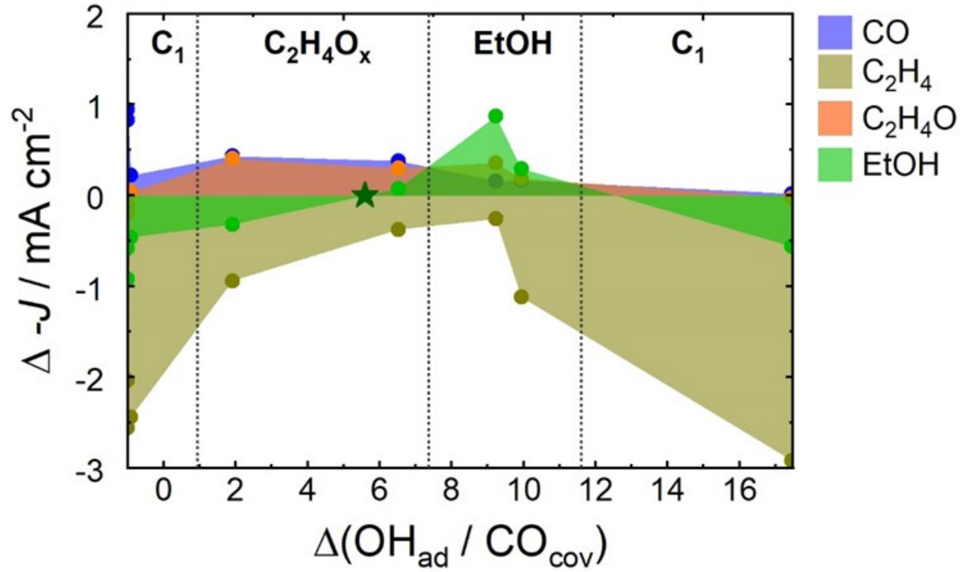

Supplementary Figure 19. Correlations between the change of the current density J of selected products (CO, ethylene, acetaldehyde, and ethanol) and the Δ (OH_{ad} / CO_{cov}) under pulsed CO_2RR conditions after subtracting the corresponding values under static CO_2RR conditions at -1.0 V. The green star represents the change of ethanol selectivity during pulsed CO_2RR up to non-oxidizing potentials at $E_a = 0$ V. The current densities were taken from our previous work.[5]

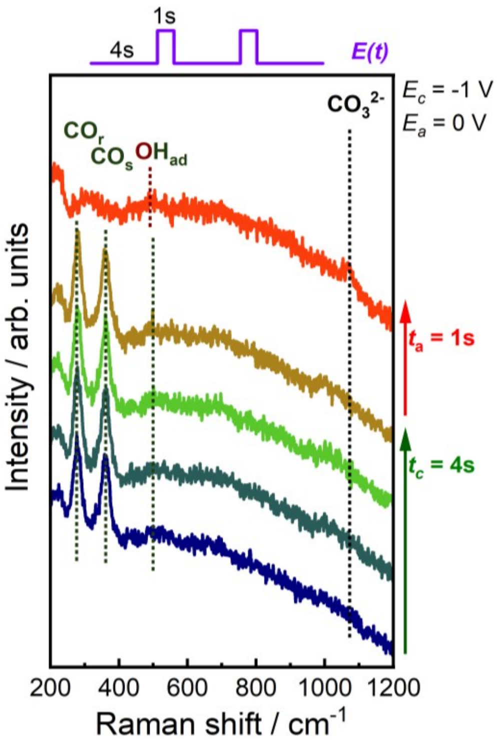

Supplementary Figure 20. Normalized SERS spectra of pulsed potential CO_2RR measurements during E_c = -1.0 V and E_a = 0.0 V with the pulse lengths of t_c = 4 s, t_a = 1 s from bottom to top (as indicated with arrows) averaged over one pulse sequence. Characteristic SERS bands are highlighted.

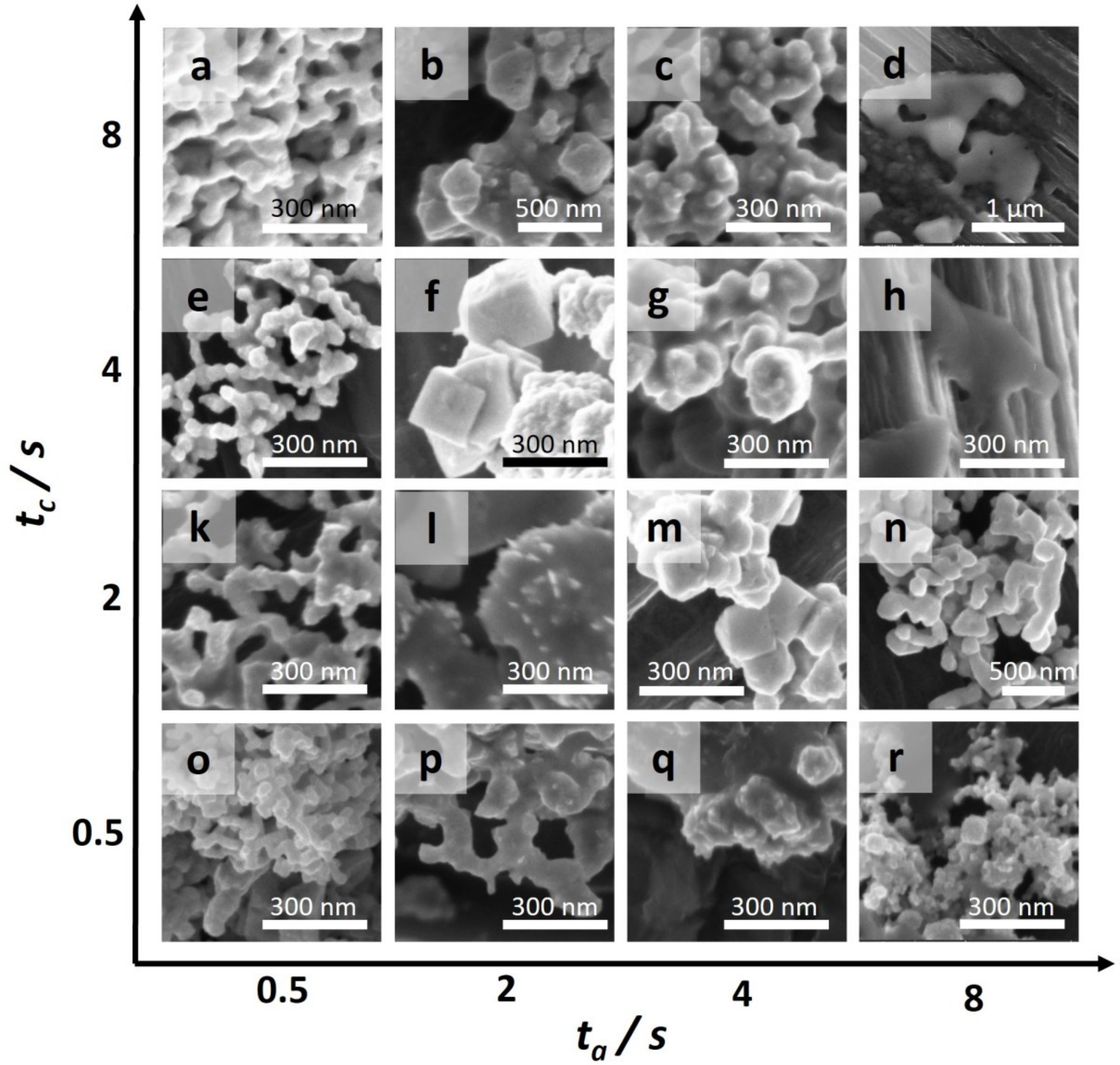

Supplementary Figure 21. Morphological evolution of samples after pulsed CO_2RR with different pulse lengths at E_c = -1.0 V and E_a = 0.6 V using ex situ scanning electron microscopy.

Supplementary Tables

Supplementary Table 1. Acquisition time of individual spectra for different pulse lengths in the lower Raman shift region of 55-1272 cm^{-1} at E_c = -1.0 V and E_a = +0.6 V, including the dead time of the spectrometer (~0.1 s).

t_c [s]	t_a [s]	Time/spectrum [s]
4	0.5	0.2
4	4	0.9
0.5	8	0.2
8	1	0.35
4	2	0.5
1	1	0.3
4	8	0.9
1	32	0.3

Supplementary Table 2. Averaged SERS band intensity values and standard derivation (arb. units) of the characteristic SERS bands over a cathodic or/and anodic pulse extracted from Fig. 2d-f and Supplementary Fig. 9.

t_c [s]	t_a [s]	CO_r (t_c)	CO_s (t_c)	OH_{ad} (t_c)	OH_{ad} (t_a)	Cu-O_{ad} (t_a)	Cu_2O (t_a)
**4**	**0.5**	0.76(9)	0.4(1)	0.20(8)	0.24(1)	0.20(5)	0
**4**	**4**	0.39(8)	0.7(2)	0.18(7)	0.25(7)	0	0.5(1)
**0.5**	**8**	0.15(5)	1.1(2)	0	0	0	0.8(1)
**8**	**1**	1.1(4)	0.6(4)	0.2(1)	0.3(1)	0	0.1(1)
**4**	**2**	0.5(1)	0.5(2)	0.4(1)	0.22(8)	0.4(1)	0
**1**	**1**	0.5(2)	0.4(1)	0.6(3)	0.4(2)	0.10(1)	0
**4**	**8**	0.22(6)	1.3(2)	0.021(9)	0	0	0.8(1)
**1**	**32**	0.33(1)	1.08(6)	0	0	0	0.6(2)

Supplementary Table 3. Time constants τ of evolution and disappearance of the CO_r and CO_s bands in arb. units during the cathodic and during the anodic pulse, respectively, extracted from the exponential decay fits in Fig. 2d-f and Supplementary Fig. 9. R^2 values indicate the goodness of the exponential fits.

t_c [s]	t_a [s]	CO_r (t_c)	R^2	CO_r (t_a)	R^2	CO_s (t_c)	R^2	CO_s (t_a)	R^2
**4**	**0.5**	0.12	0.55	0.24	0.71	0.11	0.20	0.10	0.48
**4**	**4**	0.44	0.54	0.27	0.75	0.54	0.61	0.38	0.70
**0.5**	**8**	0.043	0.50	0.04	0.29	0.08	0.75	0.08	0.76
**8**	**1**	0.20	0.29	0.05	0.22	0.45	0.20	0.13	0.20
**4**	**2**	0.13	0.47	0.35	0.69	0.24	0.58	0.53	0.77
**1**	**1**	0.23	0.26	0.28	0.29	0.23	0.28	0.41	0.34
**4**	**8**	0.51	0.20	0.01	0.77	0.57	0.84	0.36	0.90
**1**	**32**	0.03	0.25	0.02	0.77	0.06	0.20	0.02	0.52

Supplementary Table 4. Time constants τ of the disappearance and evolution of the Cu-O_{ad} and Cu_2O bands in arb. units during the cathodic and during the anodic pulse, respectively, extracted from the exponential decay fits in Fig. 2d-f and Supplementary Fig. 22. R^2 values indicate the goodness of the exponential fits.

t_c [s]	t_a [s]	Cu-O_{ad} (t_c)	R^2	Cu-O_{ad} (t_a)	R^2	Cu_2O (t_c)	R^2	Cu_2O (t_a)	R^2
**4**	**0.5**	0.04	0.20	0.11	0.20				
**4**	**4**					0.35	0.71	1.60	0.58
**0.5**	**8**					0.07	0.74	0.14	0.70
**8**	**1**					0.04	0.20	0.04	0.20
**4**	**2**	0.25	0.69	1.57	0.39				
**1**	**1**	0.02	0.67	0.41	0.20				
**4**	**8**					0.38	0.90	0.33	0.69
**1**	**32**					0.05	0.25	0.28	0.39

Supplementary Table 5. Averaged SERS band intensity values and standard derivation (in arb. units) of Cu_2O bands at 410, 530 and 620 cm^{-1} over an averaged anodic pulse and their ratios to each other.

t_c [s]	t_a [s]	*I* 410	*I* 530	*I* 620	Ratio 410/all	Ratio 530/all	Ratio 620/all	Ratio 410/ (530+620)
**4**	**4**	0.17(9)	0.33(7)	0.7(1)	0.15	0.28	0.57	0.17
**4**	**8**	0.28(7)	0.67(9)	0.9(2)	0.15	0.36	0.49	0.18
**0.5**	**8**	0.22(7)	0.8(1)	0.9(3)	0.12	0.41	0.47	0.14
**1**	**32**	0.10(4)	0.5(1)	0.8(1)	0.07	0.39	0.54	0.08

Supplementary Table 6. Change of Faradaic efficiencies (ΔFE) under pulsed conditions (-1.0 V/0 V and 4 s/1 s) subtracted by static CO_2RR conditions at -1.0 V.

**Product**	**ΔFE [%]**
H_2	-6.0(5)
CO	1.5(3)
CH_4	0.5(2)
C_2H_4	0.761(6)
$HCOO^-$	1.3(2)
CH_3COO^-	0.018(6)
Acetaldehyde	0.31(7)
Propionaldehyde	0.55(4)
Acetone	0.03(8)
Ethanol	0.76(1)
1-Propanol	0.8(1)
Allyl alcohol	0.29(4)

References

1. Zhan, C.; Dattila, F.; Rettenmaier, C.; Bergmann, A.; Kühl, S.; García-Muelas, R.; López, N.; Roldan Cuenya, B.; Revealing the CO Coverage-Driven C–C Coupling Mechanism for Electrochemical CO2 Reduction on Cu2O Nanocubes via Operando Raman Spectroscopy, *ACS Catal.* **2021**, *11* (13), 7694-7701.

2. Herzog, A.; Bergmann, A.; Jeon, H. S.; Timoshenko, J.; Kühl, S.; Rettenmaier, C.; Lopez Luna, M.; Haase, F. T.; Roldan Cuenya, B.; Operando Investigation of Ag-Decorated Cu2O Nanocube Catalysts with Enhanced CO2 Electroreduction toward Liquid Products, *Angew. Chem. Int. Ed.* **2021**, *60* (13), 7426-7435.

3. De Ruiter, J.; An, H.; Wu, L.; Gijsberg, Z.; Yang, S.; Hartman, T.; Weckhuysen, B. M.; van der Stam, W.; Probing the Dynamics of Low-Overpotential CO2-to-CO Activation on Copper Electrodes with Time-Resolved Raman Spectroscopy, *J. Am. Chem. Soc.* **2022**, *144* (33), 15047-15058.

4. Moradzaman, M.; Mul, G.; In Situ Raman Study of Potential-Dependent Surface Adsorbed Carbonate, CO, OH, and C Species on Cu Electrodes During Electrochemical Reduction of CO2, *ChemElectroChem* **2021**, *8* (8), 1478-1485.

5. Timoshenko, J.; Bergmann, A.; Rettenmaier, C.; Herzog, A.; Arán-Ais, R. M.; Jeon, H. S.; Haase, F. T.; Hejral, U.; Grosse, P.; Kühl, S.; Davis, E. M.; Tian, J.; Magnussen, O.; Roldan Cuenya, B.; Steering the structure and selectivity of CO2 electroreduction catalysts by potential pulses, *Nat. Catal.* **2022**, *5* (4), 259-267.
